# LATS2 degradation promoted fibrosis damage and rescued by vitamin K3 in lupus nephritis

**DOI:** 10.1186/s13075-024-03292-y

**Published:** 2024-03-09

**Authors:** Chen Cheng, Hao Yang, Chan Yang, Juan Xie, Jinshen Wang, Luping Cheng, Jianfu He, Honglian Li, Haoxing Yuan, Fangfang Guo, Minmin Li, Shuwen Liu

**Affiliations:** 1 Guangdong Provincial Key Laboratory of New Drug Screening, NMPA Key Laboratory of Drug Metabolism Research and Evaluation, School of Pharmaceutical Sciences, Southern Medical University, Guangzhou, 510515 China; 2 Center of Clinical Laboratory, The First Affiliated Hospital of Jinan University, Guangzhou, 510630 China; 3 State Key Laboratory of Organ Failure Research, Guangdong Provincial Institute of Nephrology, Southern Medical University, Guangzhou, 510515 China; 4 Innovation Center for Medical Basic Research On Inflammation and Immune Related Diseases, Ministry of Education, Guangzhou, 510515 China

## Abstract

**Background:**

Lupus nephritis (LN) is the most common complication of systemic lupus erythematosus (SLE). The limited treatment options for LN increase the economic burdens on patients. Because fibrotic progression leads to irreversible renal damage in LN patients and further progresses to chronic kidney disease (CKD) and the end stage of renal disease (ESRD), developing new targets to prevent LN fibrotic progression could lead to a feasible treatment strategy for LN patients.

**Methods:**

In this study, we examined YAP activation and LATS2 downregulation in LN kidney biopsy samples (LN: *n* = 8, normal: *n* = 2) and lupus-prone MRL/lpr mice (*n* = 8 for each disease stage). The function of LATS2 was further investigated by in situ injection of Ad-LATS2 into mice with LN (*n* = 6 mice per group). We examined the role of SIAH2-LATS2 regulation by IP-MS and co-IP, and the protective effect of the SIAH2 inhibitor was investigated in mice with LN.

**Results:**

Restoring LATS2 by an adenovirus in vivo alleviated renal fibrotic damage in mice with LN. Moreover, we found that LATS2 was degraded by a K48 ubiquitination-proteasome pathway mediated by SIAH2 and promoted YAP activation to worsen fibrosis progression in LN. The H150 region of the substrate binding domain (SBD) is an important site for SIAH2-LATS2 binding. The SIAH2-specific inhibitor vitamin K3 protected against LN-associated fibrotic damage in vivo.

**Conclusion:**

In summary, we identified the SIAH2-LATS2 axis as an attractive intervention target in LN to alter the resistance to fibrosis.

**Supplementary Information:**

The online version contains supplementary material available at 10.1186/s13075-024-03292-y.

## Introduction

Systemic lupus erythematosus (SLE) is an autoimmune disease with an unknown cause, various clinical manifestations, and multiorgan involvement [1, 2]. Lupus nephritis (LN) occurs in more than 60% of SLE patients during disease progression and leads to a decrease in quality of life [3]. Lupus nephritis is a major risk factor for overall morbidity and mortality in patients with SLE, and despite potent anti-inflammatory and immunosuppressive therapies, nearly 30% of LN patients still experience chronic kidney disease (CKD) or the end stage of renal disease (ESRD) [4, 5]. Thus, identifying new targets and developing specific agents to alleviate LN progression are urgently needed.

Tubulointerstitial injury is an important factor in LN-associated pathological changes [6], and renal tubular epithelial cells (RTECs) are primary targets for alleviating renal damage in LN [7, 8]. RTECs participate in LN progression in various ways, including through cytokine production, immunological activation, and fibrosis induction [9]. Tubular epithelial cell injury is a vital cause of irreversible renal fibrosis [10], which contributes to the production of ECM-related proteins, such as type I collagen and connective tissue growth factor (CTGF) [11, 12]. The promotion of tubulointerstitial fibrosis causes further RTEC death, such as CKD or ESRD progression. Renal fibrosis is an important step in the progression of immune nephritis to irreversible renal damage during LN [13]. Investigating the 2 molecular mechanisms involved in LN tubulointerstitial damage is important for developing new strategies for LN interventions [14].

Dysregulation of the Hippo pathway is involved in many diseases, including cancer, cardiovascular diseases, wound healing, and fibrosis [15]. Many studies indicate that YAP is involved in various fibrogenesis-related diseases. Connective tissue growth factor (CTGF), which is the downstream gene of YAP, is one of the most promising targets in for treating fibrosis [16]. YAP was reported to be a key factor in acute kidney injury (AKI) [17] and diabetic renal interstitial fibrogenesis [18], but the role of the Hippo pathway in LN disease progression is still unclear.

Herein, we found that YAP was activated in LN biopsy samples. The central event in Hippo pathway activation is the phosphorylation of YAP, which is regulated by large tumour suppressor kinase 1 and 2 (LATS1/2) [19]. We found that LATS2 but not LATS1 was significantly downregulated in LN. Activation of YAP was associated with LN fibrosis and renal damage. A reduction in LATS2 in renal tubular cells was also observed in mice with LN before fibrosis occurred. In vitro, knockdown of LATS2 resulted in YAP activation and increased CTGF production. Overexpression of LATS2 alleviated renal damage and interstitial fibrosis in vitro and in vivo. While the mRNA level of LATS2 did not significantly change during LN progression, we found that the downregulation of LATS2 was related to ubiquitin‒proteasome degradation mediated by seven in absentia homologues 2 (SIAH2). Moreover, we found that the SIAH2 inhibitor vitamin K3 could ameliorate the downregulation of LATS2 and fibrotic progression in mice with LN. In summary, we first report the role of the SIAH2/LATS2 axis in fibrosis damage in LN and provide new clues for long-term LN treatment and disease intervention.

## Materials and methods

### Patients

The study was proved by the Ethics Committee of Jinan University First Affiliated Hospital (approval number: KYk-2022–013). Serum was collected from patients and healthy controls. Clinical and kidney histopathological data of patients with renal biopsies proven LN diagnosis were collected (*n* = 8 patients, of whom 2 had class III LN. 4 had class IV LN and 2 had class IV plus class V LN). All patients included fulfilled the 2003 American College of Rheumatology revised criteria for SLE and diagnosed with LN by renal biopsies.

### Mice

Female MRL/lpr mice were purchased from SLAC Co. LTD (China). Age and sex matched C57/BL6 were purchased from SeBiona Biotech (China) and used as wildtype control in animal study, which is widely used to obtain physiological indicators of normal mice [20]. All mice were maintained under specific pathogen-free (SPF) conditions. All protocols of animal research in compliance with ethical regulations for animal experiments of Southern Medical University (approval number: SMUL2022326).

### Animal design

SIAH2 inhibitor vitamin K3 (cat#T0449, Top-science, USA) were dissolved in dimethyl sulfoxide (DMSO, Sigma, USA), the concentration of DMSO was maintained under 0.5% and the final concentration was 0.5 mg/ml in β-cyclodextrin (Sigma, USA). DMSO diluted in β-cyclodextrin (0.5%) was used for vehicle control, 2 mg/kg PNS was used for positive control. For vitamin K3, 2 mg/kg (low dose) and 10 mg/kg (high dose) groups were set. Of note, 10 mg/kg is a safe dose of vitamin K3 in animal experiments without causing apoptosis [21]. Adenovirus for LATS2 overexpression (Ad-LATS2-Flag) and control adenovirus (Ad-NC-Flag) were purchased from Obio Technology (China).

To investigate changes in LATS2 in the cortex during LN progression, MRL/lpr mice at different stages (*n* = 8 for each) were sacrificed. To detect the renal protective effect of vitamin K3 in LN mice, MRL/lpr mice were divided into vehicle control (Vehicle), vitamin K3 low dose group (2 mg/kg, VK3-L), vitamin K3 high dose group (10 mg/kg, VK3-H), and prednisone acetate (2 mg/kg, PNS) group (*n* = 8 for each group). C57/BL6 were served as normal control (WT) in animal experiments. For adenovirus infection, MRL/lpr mice were divided into sham group, Ad-vehicle group and Ad-LATS2 group (*n* = 6 for each group). Adenovirus was injected in situ through the kidney. The kidneys were sampled at the indicated time, renal tissues were divided longitudinally into two parts. One was collected for RNA and protein extraction and another was fixed with 4% paraformaldehyde and embedded in paraffin.

### Biochemical analysis of mice urine

Mice 24 h proteinuria was collected by metabolic cage. The levels of urinary protein and creatinine were analyzed by urine protein quantitative test kit (CBB method, Jiancheng, China) and creatinine quantitative test kit (Jiancheng, China).

### Cell culture and treatments

HEK293T and HK-2 cells were purchased from American Type Culture Collection (ATCC, USA). Both were cultured in DMEM (Gibco, USA) supplemented with 10% FBS (Excell Bio, China) and 1% penicillin (100 units/ml) /streptomycin (100 μg/ml) (Gibco, USA). Fresh medium was replaced before transfection and the plasmids were transfected with lipofectamine 3000 (Thermo-fisher, USA) according to the manufacturer’s instruction. 8 ng/ml TGF-β (Peprotech, USA) was used to treat HK-2 cells to construct tubular fibrosis model in vitro. Treatment with anti-dsDNA Ab was consistent with the previous study, additional anti-dsDNA Ab was required while fresh culture exchanged [22].

### Knockdown and overexpression

Small interfering RNAs (siRNAs) targeting LATS2 (si-LATS2), SIAH2 (si-SIAH2), and nontargeting control siRNA (si-NC) were synthesized at Gene-Pharma (China). The pCMV-Flag-LATS2 and pCMV-Zyxin plasmids were constructed in our laboratory. The shYAP HK-2 cell line was constructed in our laboratory by using a Tet-on lentivirus system. All cell transfections with siRNA were conducted with Lipofectamine 3000 (Thermo-Fisher Scientific), following the manufacturer’s instructions.

### Renal histology and immunohistochemical staining

The Sects. (4 μm thickness) after deparaffinization were stained with hematoxylin and eosin to observe structural characteristics of renal tissues. Masson and Sirus-red were performed to reflect the deposition of collagens. For immunohistochemical staining, the deparaffinized sections were put in microwave oven to repair antigen. Slides were permeated in 0.1% tritonx-100 and then incubated with anti-LATS2 (Novus, USA), anti-YAP (CST, USA), anti-αSMA (Protein-tech, USA), anti-SIAH2 (Novus, USA), anti-Zyxin (Santacruz, USA) and anti-CTGF (Santacruz, USA) at 4℃ overnight and cover with endogenous peroxidase blockers before incubating with corresponding HRP-conjugated secondary antibodies (Fude-biological, China). Slides were stained with 3,3′-diaminobenzidine (DAB) and then imaged under microscope (Zeiss, Germany).

### Confocal microscope

Tissue sections were stained with antibodies to LATS2 (Novus, USA), SIAH2 (Novus, USA), CTGF (Santa-Cruz, USA), YAP (CST, USA) and α-SMA (Protein-tech, USA) for 14–16 h at 4℃ and followed by staining with Alexa-488 conjugated anti-Rabbit and Alexa-595 conjugated anti-Mouse respectively. 4’, 6-diamidino-2- phenylindole dihydrochloride (DAPI) was used to color nuclei. Confocal images were recorded by FV3000 confocal microscope (Olympus, Japan).

### RNA isolation quantitative polymerase chain reaction (qPCR)

Total RNA was lysed and collected by total RNA isolation kit (Foregene, China). The RNA of mouse kidney cortical tissues was isolated and lysed in pulp refiner (K-II, Service-bio, China). Reverse transcription kit (Takara, Japan) was used to synthesis first cDNA strand. All reactions were performed with Syber Green I Master (Avid-biotech, China) according to the manufacture’s introductions. All samples were finally amplificated and analysis in 2 ^−(ΔΔCq)^ way by using Light Cycler 480 (Roche, Germany). Primers used in this study were provided in Supplementary Table 1.

### Detection of ANA

Anti-nuclei antibody (ANA) level was detected by indirect immunofluorescence assay. HK-2 cells were seeded in 12-well plate at 10^5 cells/ml. Cells were fixed with 4% paraformaldehyde and permeated in 0.1% tritonx-100. Mouse serum was collected and diluted in PBS for 20 times dilution then incubated with fixed HK-2 cells overnight. Alexa-488 conjugated anti-mouse IgG (Abcam, UK) was used to detect mouse anti-nuclei antibody level.

### Western blot

Cells or renal cortical tissues were lysed in cold RIPA lysis buffer (Thermo Fisher, USA) supplemented with protease and phosphatase mixture inhibitor (Sigma, USA). Protein concentration was measured by BSA assay. The whole lysates were denatured with 1 × loading buffer in 95℃ for 10 min and then separated by 10% SDS page. Blotting onto nitrocellulose membranes was performed in wet transfer assay. The primary antibodies followed by corresponding secondary antibodies (dilution 1:5000) were used and finally visualized with Fluor Chem E device (Protein Simple, USA).

### Co-IP and IP-MS (mass spectrum)

HEK-293 T cells were seeded in dishes at 4 × 10^5 cells/ml 24 h before the Flag-LATS2 and HA-Ub plasmids transfection. After 48 h, the cells were lysed by IP buffer and the protein concentration was measured. The equivalent protein was mixed with pre-incubated beads for 16 h, followed by three times wash, beads was used for MS analysis supported by Wayen Biotechnologies Co., Ltd. (China). Or the protein linked on beads was finally denatured in 1 × sample buffer at 95℃ for 10 min and western blot assay was performed subsequently.

### Dual-luciferase assay

pGL3-basic is a reporter plasmid encoding luciferase gene, lack of gene-promoter. The promoter of SIAH2 was constructed in pGL3-basic and named pGL3-SIAH2p. pCMV-RUNX1-Flag was maintained in our laboratory. HEK-293 cells were seeded in a 12-well plate at 200,000 cells per well 1 day before transfection and then transfected with 250 ng pGL3-basic or pGL3-SIAH2p and 100 ng pCMV-RUNX1-Flag by using polyjet (Signa-Gen, USA). After 48 h, the cells were harvested by 250 μL lysis buffer and the Dual-Luciferase Reporter Assay System (Promega, USA) was used to detect the fluorescence activity. Each sample was set in triplicate holes and the experiment was repeated twice.

### Chromatin Immunoprecipitation (Ch-IP) qPCR

HEK293T cells were fixed with 1% (wt/vol) formaldehyde and subsequently quenched with glycine. Ch-IP qPCR was performed according to manufacturer’s instructions (Wanlei Bio, China). Briefly, the fixed samples were suspended in cell lysis buffer and collected nuclei were stored at -80 °C in lysis buffer. For RUNX1 Ch-IP, lysates were thawed and sonicated with HX-1800E (Huxi, China) to obtain chromatin fragments of 300–1,000 bp, and tenfold-diluted with Ch-IP dilution buffer. After washing, a complex of nuclear proteins/DNAs and antibodies against Flag was retrieved with Protein A/G Dynabeads (Wanlei Bio, China). After the cross-linking was reversed, chromatin fragments were treated with RNase A and proteinase K. DNA was purified for Ch-IP qPCR analysis (Wanlei Bio, China)., the values from the immunoprecipitated samples were normalized to that from the input DNA. Primer sequences are available upon request.

### Enzyme linked immunosorbent assay (ELISA)

Mice renal tissues of equal weight were collected and homogenized in EDTA anticoagulated tubes, and the supernatant was separated after centrifugation at 4000 rpm/min for 15 min. The mice renal TGF-β and mice serum anti-dsDNA antibody were detected by ELISA kits (Meimian, China, cat#MM-0135M1 and cat#MM-45766M1) according to the instructions.

### Docking analysis

The 3D crystal structure of the SIAH2 protein was downloaded from the RCSB Protein Data Bank (www.rcsb.org) using the PDB ID of 3EYG, and water and glycosyl molecules were removed manually. A molecular docking study was performed using Discovery Studio 3.0. A grid for the docking simulations was generated with the default setting by centering it based on the co-crystallized ligand MI1. Molecular docking is performed using an extra precision (XP) docking mode, in which the receptor remains rigid and the ligand is free to move.

### Data acquisition and analysis

The mRNA expression profiles GSE127797 were downloaded from NCBI Gene Expression Omnibus (GEO, https://www.ncbi.nlm.nih.gov/geo/). We extracted tubular mRNA profile (normal:15, LN:32) from GSE127797 by using the Practical Extraction and Report Language (Perl). The heatmap and volcano map of data were visualized by R (4.1.1). Packages “limma” and “pheatmap” were used, and log|Fc|> 2 and *p* < 0.05 were filter conditions.

### Statistical analysis

All data examined are expressed as mean ± SEM. Statistical analyses of the data were performed using Graphpad Prism (Version 8.0). The comparisons between groups were analyzed using one-way ANOVA. *P* < 0.05 was considered to have a significant difference (*p < 0.05, **p < 0.01). Semi-quantitative analysis was performed by Image J.

## Results

### The Hippo pathway is involved in LN

**Fig. 1:**
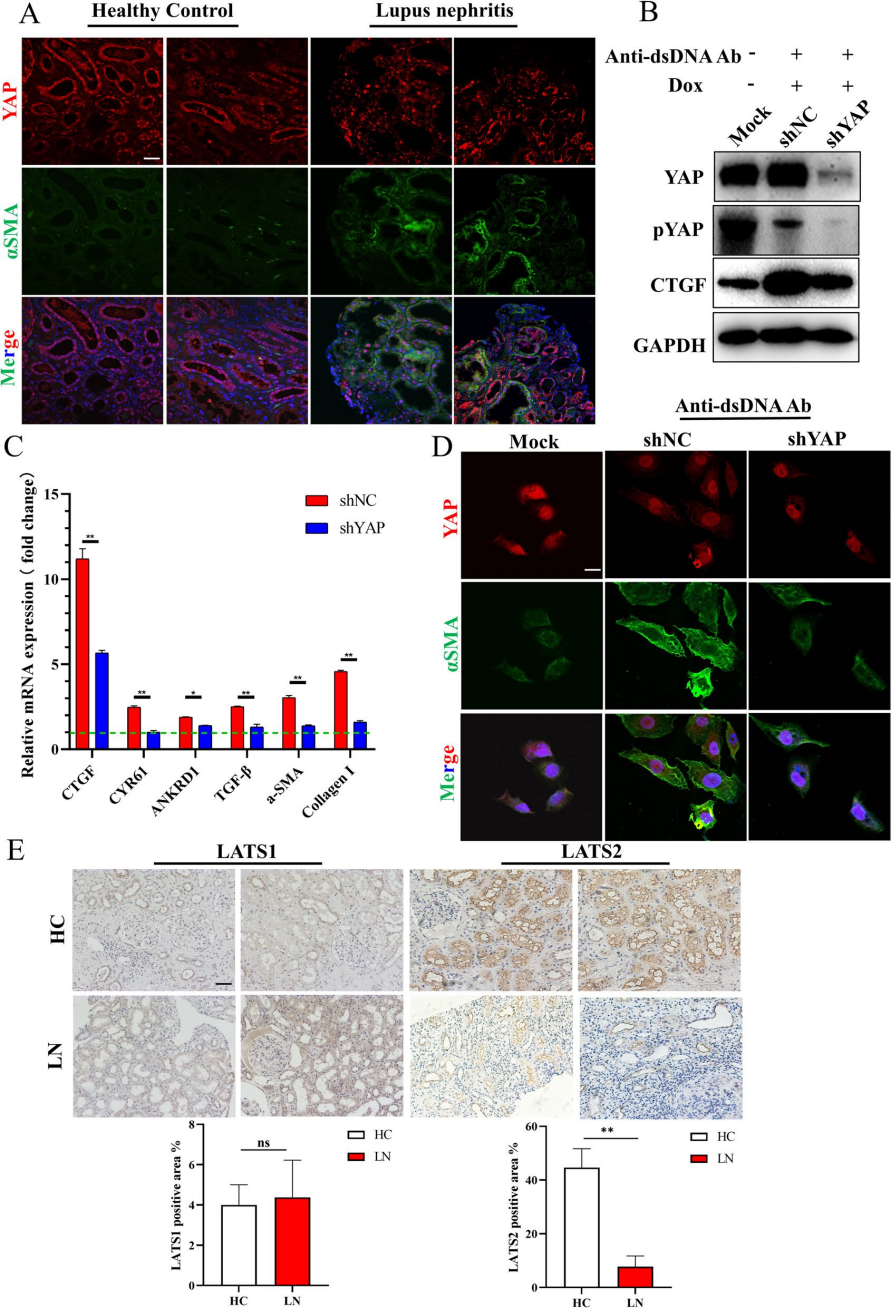
Hippo pathway was involved in LN progression. **A**, YAP activation was found in LN biopsies and nuclear transduction was correlated with fibrotic factors α-SMA (scale bar, 20 μm). **B**, Knockdown of YAP alleviated the CTGF production induced by Anti-dsDNA Ab. **C,** Knockdown of YAP reduced profibrotic genes expressions induced by Anti-dsDNA Ab. **D**, Anti-dsDNA Ab promoted YAP nuclear transduction and fibrotic progression (scale bar, 2 μm). **E**, LATS2 but not LATS1, was downregulated in LN tubulars (scale bar, 20 μm)

We examined the localization of YAP, which is a core factor in the Hippo pathway, in LN biopsy samples. We found that YAP was mainly located in the proximal tubular nucleus and was associated with increased production of α-SMA (Fig. 1A). Anti-dsDNA Ab is a unique and important pathogenic molecule in LN, and we found that treatment with anti-dsDNA Ab in vitro caused nuclear accumulation of YAP and fibrotic transformation in HK-2 cells compared to healthy controls (Figure S1). However, YAP accumulation was not observed until the ninth day of treatment with the anti-dsDNA Ab. These findings indicated that Hippo was involved in the progression of LN associated fibrosis. To further examine the role of YAP, we generated a Dox-responsive YAP-knockdown HK-2 cell line by using the TetON-shYAP pseudo-virus system. Knockdown of YAP blocked the production of fibrotic factors induced by the anti-dsDNA Ab (Fig. 1B and C) and the progression of fibrotic characteristics (Fig. 1D). Since LATS1 and LATS2 are the major regulators of YAP, we also examined their expression in LN biopsy samples. Interestingly, the expression of LATS2 but not LATS1 was significantly downregulated in lupus nephritis specimens, especially in proximal tubules (Fig. 1E). However, how LATS2 is degraded and whether this degradation is related to YAP activation and LN fibrosis have not been reported.

### LATS2 is downregulated during LN progression

**Fig. 2:**
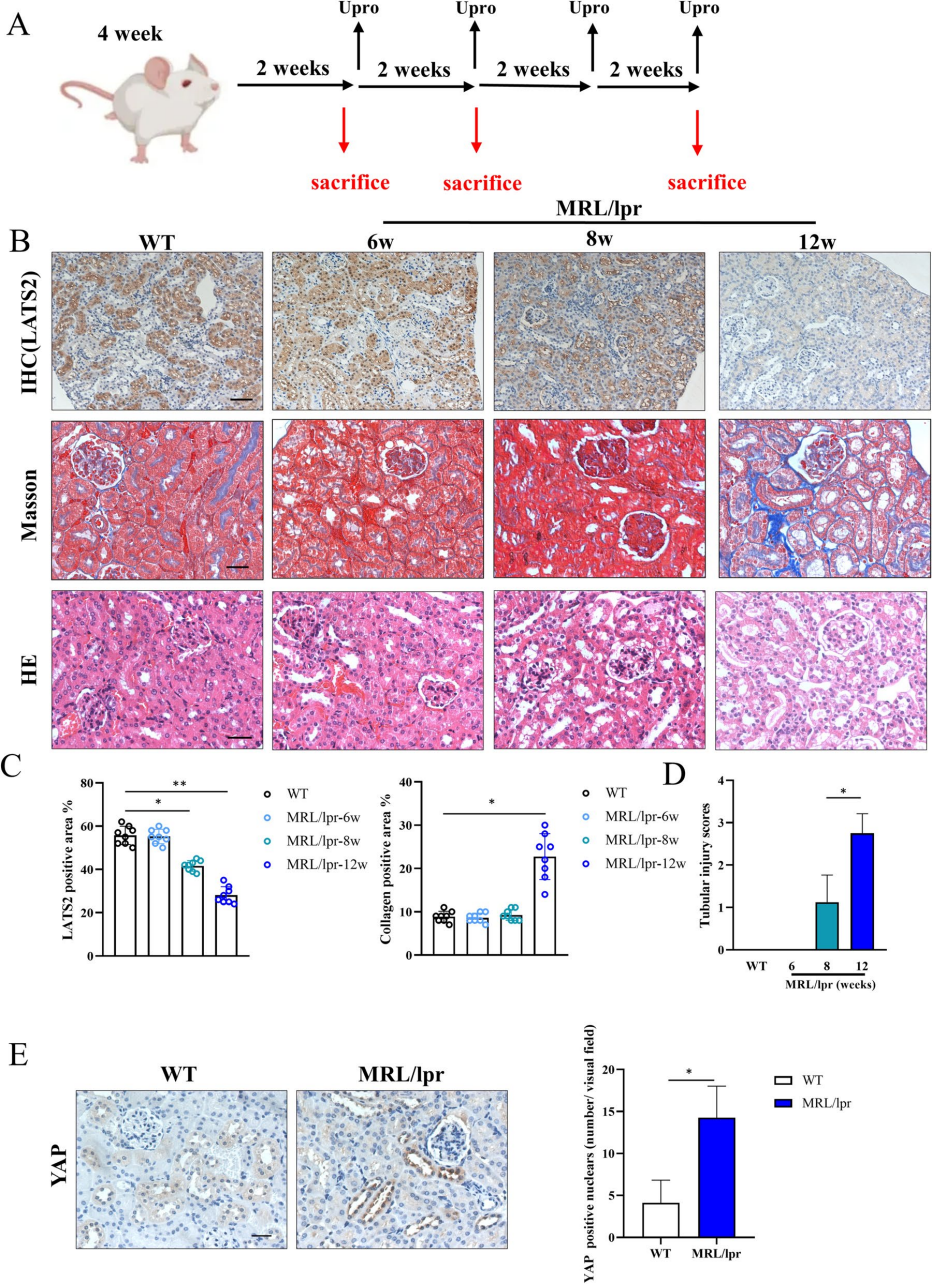
LATS2 was downregulated in LN progression. **A**, Scheme of lupus mice sacrifice and proteinuria collection in different disease stage. **B**, Top: IHC staining of LATS2 in different stage of lupus mice (scale bar, 40 μm). Middle: Masson staining in different stage of lupus mice (scale bar, 20 μm). Bottom: HE staining in different stage of lupus mice (scale bar, 20 μm). **C**, Expression of LATS2 intensity by grayscale analysis in lupus mice (*n* = 8 per group). Left, 6-week. Middle, 8-week. Right, 12-week. **D**, Score of tubular injury in different stage of lupus mice (*n* = 8 per group). **E**, YAP localization in LN mice (scale bar, 20 μm)

**Fig. 3:**
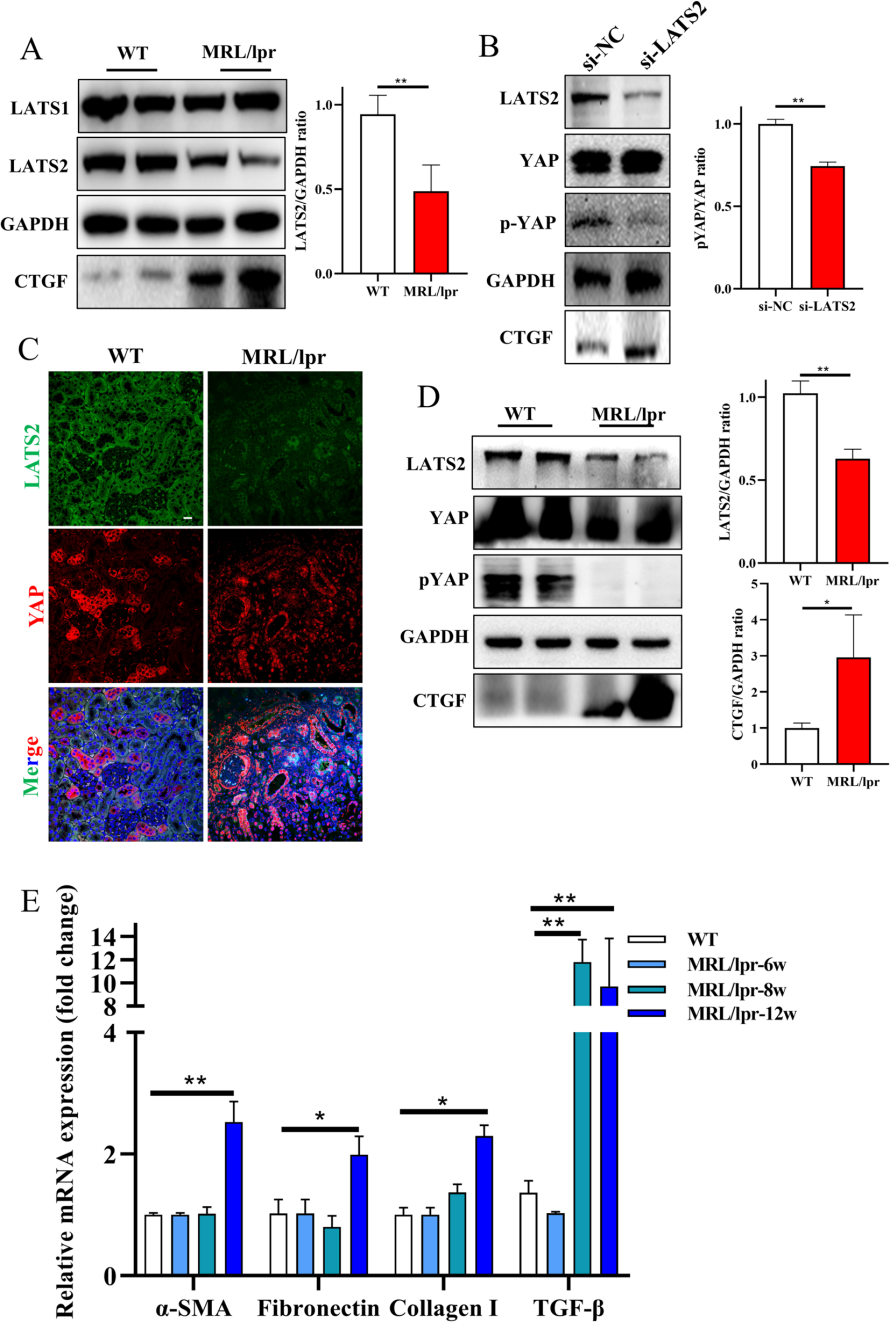
Reduction of LATS2 promoted YAP activation and CTGF production in vitro and in vivo. **A**, LATS2, instead of LATS1, was down-regulated in renal cortex in LN mice. **B**, Knockdown of LATS2 promoted YAP activation and CTGF production in HK-2 cells. **C**, Co-localization analysis of LATS2 and YAP in LN (scale bar, 20 μm). **D**, Hippo pathway in LN mice cortex analyzed by western blot. **E**, Expression of pro-fibrotic genes in different stage of lupus mice

When is the LATS2 altered, and what is the molecular role of LATS2 in LN? MRL/lpr mice were used to examine the relationship between the reduction in LATS2 expression and disease duration. Female mice are still the commonly used in mouse models of systemic lupus erythematosus and are closely related to clinical phenomena. Female MRL/lpr mice were harvested after 6, 8, or 12 weeks (Fig. 2A). The results showed increases in the albumin–creatinine ratio, anti-dsDNA Ab titer, ANA titer and renal TGF-β concentrations during LN progression (Figures S2 and S3). This finding indicated that the disease gradually worsened, and the kidney became involved after 8 weeks, as determined by renal immune complex (IC) deposition (Figure S3B). The course of lupus in MLR/lpr mice was analyzed, and mice with renal damage and IC deposition were considered mice with LN. Tubular LATS2 expression was decreased after 8 weeks in mice with LN and was significantly decreased after 12 weeks (Fig. 2B and C). Tubular dilatation and fibrosis were also more severe after 12 weeks (Fig. 2B and D). At this stage of LN, YAP was localized in the tubular nucleus (Fig. 2E). Moreover, the expression of LATS2 but not LATS1 was significantly downregulated in the kidney in LN (Fig. 3A). The production of CTGF, a profibrotic factor downstream of YAP, was significantly increased (Fig. 3A). LATS2 plays important roles in kidney development and renal cancer [23]. However, the role of LATS2 in LN-associated damage is still unknown. To understand the relationship between LATS2 downregulation and LN-associated tubular injury, we knocked down LATS2 in human tubular epithelial HK-2 cells. LATS2 knockdown promoted the dephosphorylation of YAP, causing YAP activation and increasing downstream CTGF production (Fig. 3B). LATS2 downregulation occurs early in LN renal involvement and knockdown was correlated to the activation of YAP and the progression of fibrosis injury. To demonstrate the relationship between LATS2 downregulation and the activation of YAP in LN visually, we performed confocal analysis of LATS2 and YAP in LN proximal tubulars, our results showed that phosphorylated YAP is mainly localized in the cytoplasm and was highly co-localized with LATS2 in proximal RTECs in WT. In mice with LN, the LATS2 was decreased and YAP was mainly located in the nucleus (Fig. 3C). Moreover, the production of CTGF was also increased in LN proximal tubulars and showed correlation with LATS2 downregulation (Figure S3C). We further proved the LATS2 reduction and YAP activation by western blot analysis, and decreased YAP phosphorylation was also observed in cortex of mice with LN (Fig. 3D and E). Therefore, the abnormal down-regulation of LATS2 is an important reason for the increased nuclear entry and activation of YAP in tubular cells and the production of pro-fibrotic factors in LN renal tubules.

### Restoring LATS2 levels reduces fibrosis progression in mice with LN

**Fig. 4:**
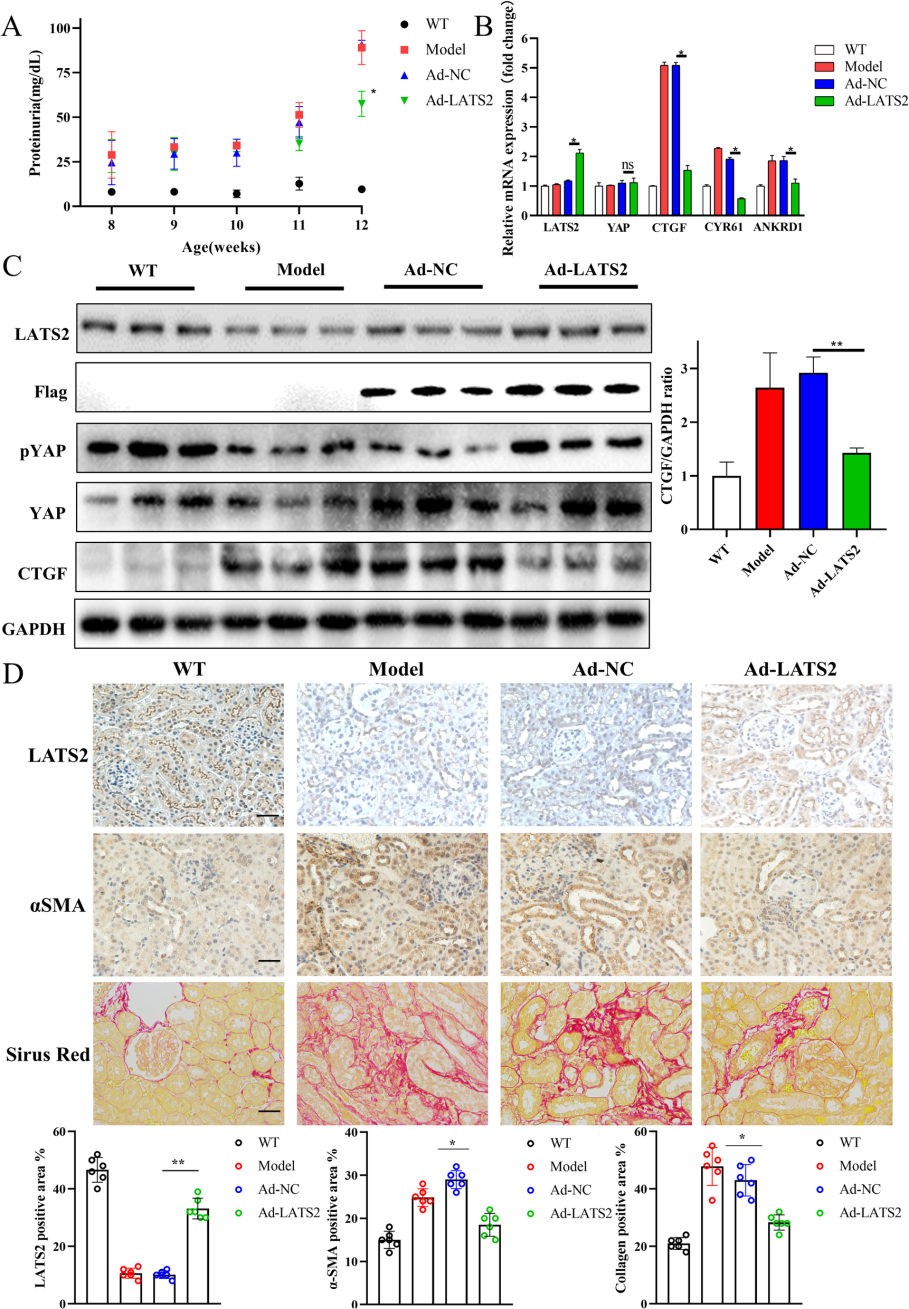
Restoring LATS2 alleviated renal damage in LN fibrotic progression. **A**, Proteinuria of LN mice (*n* = 6 per group). **B**, Expressions of YAP, and its signature downstream genes in LN mice. **C**, Western blot analysis of LATS2 and YAP in LN mice cortex. Overexpression of LATS2 reduced YAP activation and CTGF production.** D**, Top, expression of LATS2 by IHC staining. Middle, expression of a-SMA by IHC staining. Bottom, Sirus red staining (scale bar, 20 μm)

Renal fibrosis is a common pathway leading to chronic kidney disease (CKD) and is the main pathological precursor of ESRD. As irreversible kidney damage occurs, preventing fibrotic transformation and progression in LN is important. However, the mechanisms of fibrosis progression and the various causes of CKD are significantly different. To investigate the role of LATS2 in LN-associated renal damage, an adenovirus encoding LATS2 (Ad-LATS2) was constructed for further in vivo analysis. We performed in situ renal injection in MRL/lpr mice and successfully overexpressed exogenous LATS2 at both the mRNA and protein levels in vivo (Figure S4A-D). Moreover, overexpression of LATS2 did not cause proteinuria-mediated damage in mice (Figure S4E). MRL/lpr mice were further divided into the sham, Ad-vehicle, and Ad-LATS2 groups. Overexpression of LATS2 protected renal function (Fig. 4A) and increased the phosphorylation of YAP. Impairing YAP activity decreased the production of downstream genes, especially CTGF (Fig. 4B and 4C). CTGF is involved in cell proliferation, migration, and differentiation and can promote the progression of organ fibrosis. Consistently, overexpression of LATS2 decreased the expression of fibrotic indicators in cortical regions and effectively reduced the overall progression of fibrosis (Fig. 4D). The effect of Ad-LATS2 on inflammation is not as strong as it is on profibrotic factors (Figure S4F). This may be related to the heterogeneity of the model mice and the various types of kidney cells. In sum, our finding indicated that ameliorating the pathological downregulation of LATS2 is beneficial for preventing the progression of fibrosis and renal damage in LN.

### LATS2 is downregulated by SIAH2-mediated ubiquitination in LN

**Fig. 5:**
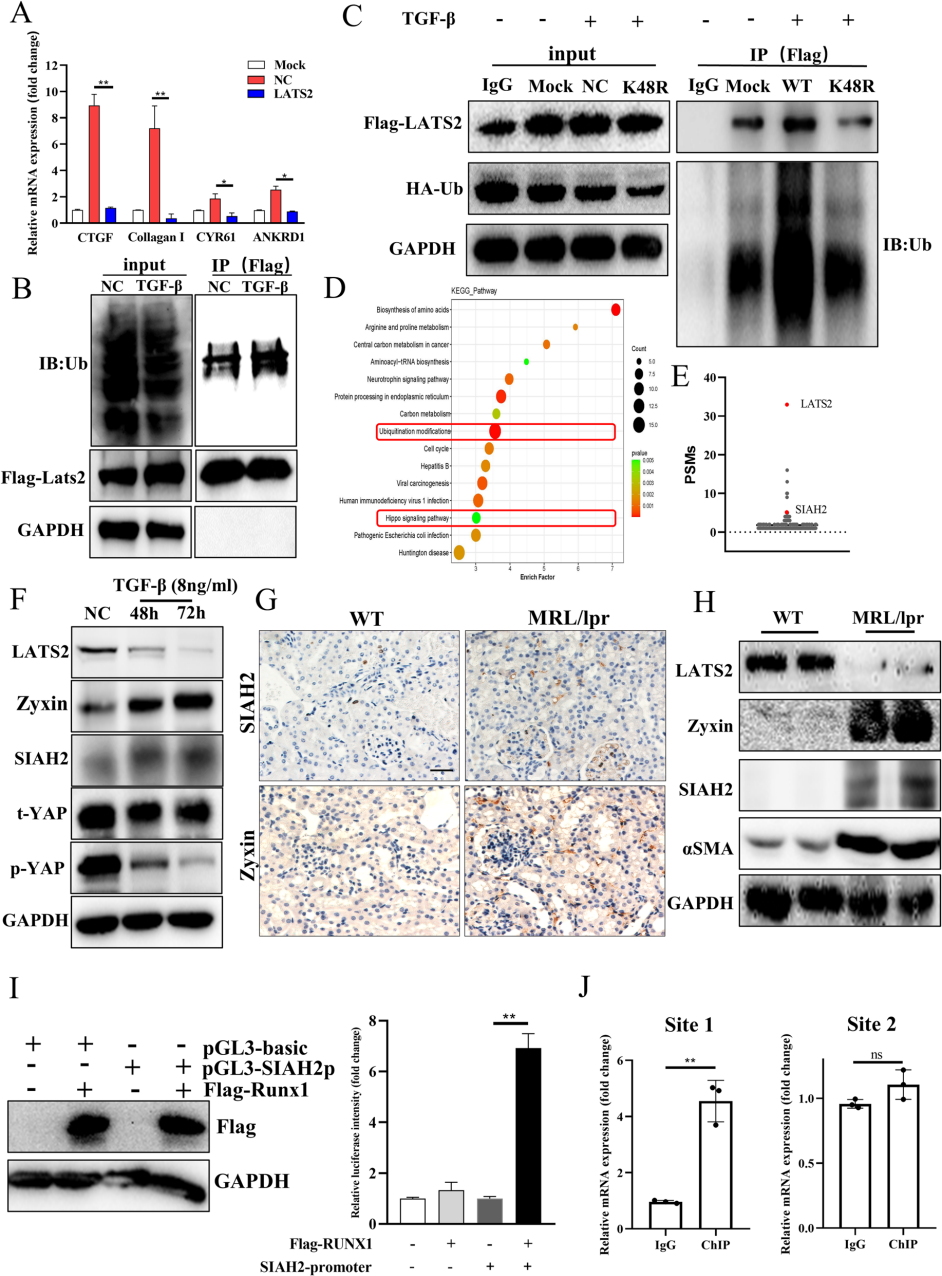
LATS2 was downregulated in K48 ubiquitin–proteasome way. **A**, Overexpression of LATS2 regulated CTGF production, and YAP downstream genes. **B**, TGF-β promoted LATS2 ubiquitination in HK-2 cells. **C**, K48R mutation of Ub protected LATS2 ubiquitinated markers in HK-2 cells. **D**, KEGG pathway analysis of interacted proteins with LATS2. **E**, PSMs of LATS2 and SIAH2 in IP-MS analysis. **F**, TGF-β treatment increased SIAH2 and promoted YAP activation in HK-2 cells.** G**, Expression of SIAH2 and Zyxin in LN mice by IHC staining (scale bar, 20 μm).** H**, Western blot analysis of SIAH2-LATS2 axis in LN mice.** I**, Dual-luciferase analysis of the interaction between RUNX1 and SIAH2-promoter, RUNX1 promoted luciferase transcription in pGL3-SIAH2p. **J**, Ch-IP qPCR analysis of the SIAH2 transcription regulated by RUNX1

Although LATS2 was significantly decreased, the mRNA level of LATS2 was not significantly changed in mice with LN (Figure S5A). Due to the lack of human kidney RNA samples, we analyzed the spatial transcriptomics data of LN and found that the mRNA level of LATS2 was also not significantly decreased in the tubules of LN patients (GSE127797, Figure S5B). As shown in Figure S1, YAP activation induced by the anti-dsDNA Ab was not observed until the 5th day. The anti-dsDNA Ab induced fibrosis mainly through TGF signaling in LN [24], and we investigated whether TGF-β was the direct factor through which the anti-dsDNA Ab caused Hippo signaling in renal cells. We found that treatment with TGF-β downregulated LATS2 in HK-2 cells in vitro, and rescuing LATS2 impaired the production of profibrotic factors induced by TGF-β; consistently, the mRNA level of LATS2 was not changed (Figs. 5A and S5C). Recently, renal single-cell sequencing of LN patients showed that ubiquitination plays an important role in LN progression [11], and we found that TGF-β increased the ubiquitination level of LATS2 (Fig. 5B), indicating that the ubiquitin‒proteasome pathway could be responsible for the downregulation of LATS2. Mutating ubiquitin at the K48 site prevented TGF-β from increasing the ubiquitination level of LATS2 (Fig. 5C). Moreover, we analyzed the binding of E3 ligases to LATS2 in HK-2 cells by IP-MS, and the results showed that LATS2 was regulated by ubiquitination (Fig. 5D). The E3 ligase SIAH2 was identified among the high peptide-spectrum matches (PSMs) and has been reported to regulate LATS2 in breast cancer (Fig. 5E). SIAH2 destabilizes LATS2 in breast cancer through hypoxia signaling [25]. Zyxin was reported to promote SIAH2-mediated LATS2 ubiquitination as a scaffold protein [26]. We found that the mRNA levels of SIAH2 and Zyxin were upregulated in the tubules of mice with LN (Figure S5D-E). We observed an interaction between LATS2 and SIAH2 by IP, which was consistent with the findings of previous reports. Interestingly, in the present study, among the four main functional amino acids in the substrate recognition region (SBD) of SIAH2, H150 was important for the recognition of LATS2. We further proved that K672 of LATS2 and H150 of SIAH2 were important for the SIAH2-LATS2 axis connection (Figure S5F-G). IHC staining revealed that the numbers of SIAH2- and Zyxin-positive cells were increased in mice with LN (Fig. 5F-G). Similarly, the expression of SIAH2 and Zyxin increased with increasing α-SMA production (Fig. 5H). Furthermore, we found that the upregulation of SIAH2 was associated with RUNX1, a transcription factor that is activated by TGF-β signaling (Supplementary Table 2). Dual-luciferase assay showed that RUNX1 increased the expression of luciferase which is the downstream of the SIAH2 promoter in pGL3-SIAH2p, suggesting that RUNX1 could bind to the SIAH2 promoter (Fig. 5I). Furthermore, Ch-IP qPCR showed that RUNX1-mediated transcription of SIAH2 accounted for a certain proportion of the total transcription at site 1 (Supplementary Table 2), could be important for the increase in SIAH2 (Fig. 5J). Hence, we demonstrated the regulatory relationship between TGF-β and Hippo through the ubiquitination of SIAH2-LATS2 in LN. TGF-β increases the transcriptional activity of RUNX1, which in turn increases the expression of SIAH2 and causes the degradation of LATS2, leading to the activation of YAP and an increase in ECM, resulting in fibrosis progression.

### The SIAH2 inhibitor vitamin K3 rescues the reduction in LATS2 and decreases RTEC damage in LN

**Fig. 6:**
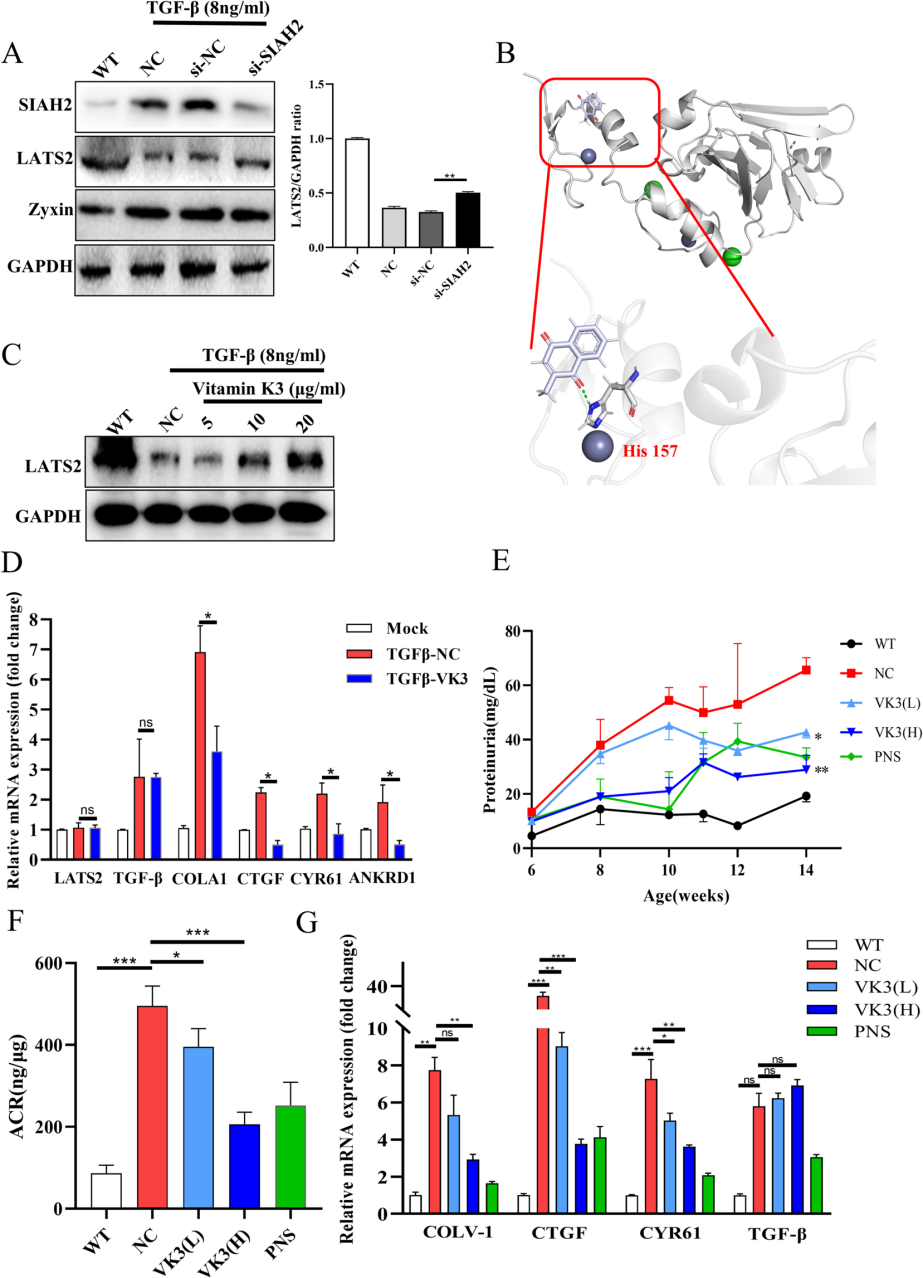
Target SIAH2 alleviated LATS2 reduction and protect renal damage in LN mice. **A**, Knockdown of SIAH2 decreased LATS2 degradation induced by TGF-β. **B**, Molecular docking analysis of vitamin K3 in SIAH2 SBD region. **C,** Vitamin K3 restored LATS2 levels in dose dependent. **D**, Vitamin K3 inhibited YAP downstream genes production induced by TGF-β.** E**, Vitamin K3 controlled the increase of proteinuria in LN mice (*n* = 8 per group). **F**, Vitamin K3 decreased the ACR in LN mice (*n* = 8 per group).** G**, Vitamin K3 reduced the collagen I and CTGF transcriptions in LN mice cortex in dose dependent

**Fig. 7:**
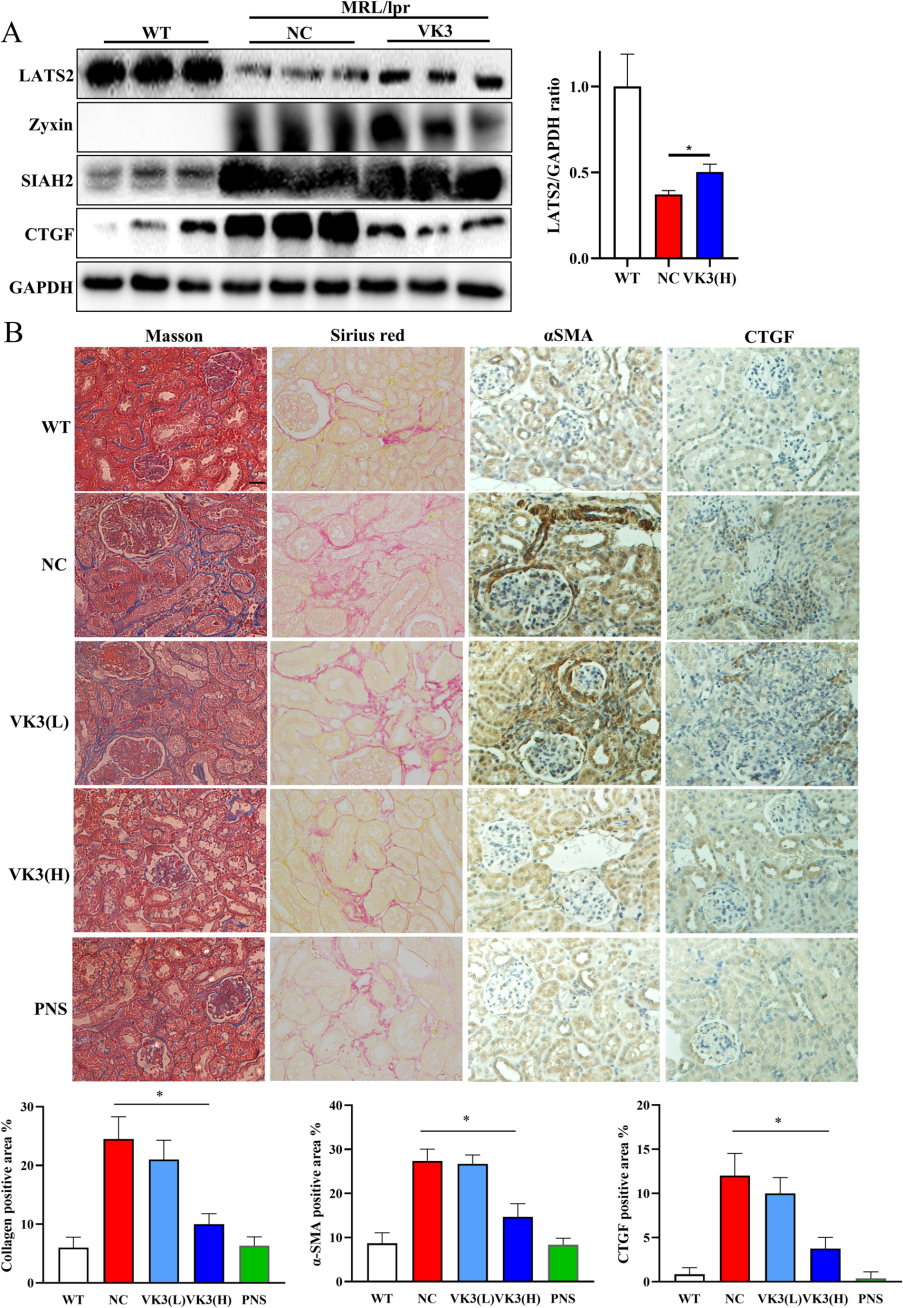
High dose administration of vitamin K3 reduced renal fibrotic damage in LN mice. **A,** Vitamin K3 (10 mg/kg) alleviated LATS2 downregulation in LN mice and inhibited CTGF production. **B**, Vitamin K3 reduced fibrotic progression and fibrotic factors production in dose dependent in LN mice (scale bar, 20 μm)

Knockdown of SIAH2 alleviated the degradation of LATS2 and the upregulation of SIAH2 induced by TGF-β (Fig. 6A). Vitamin K3, a specific inhibitor of SIAH2, was reported to ameliorate pulmonary hypertension and tumor resistance by inhibiting SIAH2 [27, 28]. Interestingly, docking analysis showed that vitamin K3 could form a hydrogen bond with His157 in the pocket near the key H150 site in SIAH2 (Fig. 6B). The in vitro results showed that vitamin K3 alleviated the downregulation of LATS2 in a dose-dependent manner and reduced YAP activation induced by TGF-β in HK-2 cells (Fig. 6C and D). We examined the protective effect of oral administration of vitamin K3 on renal damage in vivo. Moreover, vitamin K3 protected against proteinuria progression and renal dysfunction in mice with LN (Fig. 6E and F). Vitamin K3 decreased the production of profibrotic factors that are important for LN-associated fibrosis. The expression of profibrotic factors was significantly downregulated in the VK3-H group (Fig. 6G). High-dose administration of vitamin K3 ameliorated the downregulation of LATS2 in the renal cortex of mice with LN in vivo (Figs. 7A and S5H) and successfully reduced CTGF production by inhibiting SIAH2 activity (Fig. 7A). Pathological and fibrosis marker analysis showed that vitamin K3 alleviated renal dysfunction and interstitial fibrosis in a dose-dependent manner in mice with lupus (Fig. 7B). Our results suggested that rescuing LATS2 by inhibiting SIAH2 is a potential approach to prevent LN fibrosis and ESRD progression.

## Discussion

Treatment of LN typically involves immunosuppressive therapy such as mycophenolate mofetil or cyclophosphamide, as well as glucocorticoids, although these treatments are not uniformly effective [4]. Immunosuppressive therapy effectively alleviates acute renal inflammation but is not effective at preventing further nephron loss during long-term LN treatment. Clearly, investigating potential therapeutic targets and identifying reno-protective treatments are important for treating LN [16]. Our approach focuses on the long-term consequences of LN (i.e., LN fibrosis and renal function maintenance). In this study, we demonstrated the role of SIAH2-LATS2 in LN renal damage. Downregulation of LATS2 but not LATS1 promoted LN progression via CTGF and fibrotic factor production in tubules. Notably, restoring LATS2 expression by in situ injection of Ad-LATS2 or the SIAH2-specific inhibitor vitamin K3 conferred renal protection and prevented fibrotic progression in mice with LN. Thus, our data extend the current knowledge of LATS2 to the field of autoimmune disease, providing evidence that LATS2 is a promising therapeutic target in LN progression. The safety of vitamin K3 (menadione), which is a marketed drug, allows for the possibility of LN treatment in the future.

A novel finding in this study is that the Hippo pathway is involved in LN renal damage and that the SIAH2-LATS2 axis plays an important role in fibrosis progression. Kidney fibrosis initiates at certain focal sites in which the fibrogenic niche is formed [29]. Risk factors for this fibrogenic niche include fibroblast proliferation and tubular epithelial-to-mesenchymal transition. Fibrosis is a major event in the progression of LN and can occur early, requiring appropriate intervention during clinical treatment [30]. CTGF, the central profibrotic factor of the Hippo pathway, is essential for the accumulation of ECM and the progression of renal fibrosis [31]. The use of the anti-CTGF mAb to treat fibrotic diseases is already in clinical trials. However, the development of new targets for different fibrotic diseases is still urgently needed. It was recently reported that LATS2 might be involved in liver fibrosis [32], which is consistent with our results. These findings confirm that LATS2 could be a new target for fibrosis prevention. Directly targeting TGF-β may have adverse effects on many biological processes, such as apoptosis, proliferation, and immune responses, which are regulated by TGF-β [33]. The regulatory role of RUNX1-SIAH2-LATS2 was first described in the present study. We believe that targeting RUNX1 or SIAH2 may be useful for intervening in signaling crosstalk [34]. However, additional evidence is needed to support the use of RUNX1 inhibitors in to treat LN.

LN is a major complication of SLE and is present in more than 50% of SLE patients during disease progression. LN is typically associated with a poor long-term prognosis, and up to 30% of LN patients progress to CKD or ESRD, which is associated with a fourfold increase in mortality [35]. The pathogenesis of LN involves the renal microenvironment, which includes immune cells and various types of renal cells [36]. Studying the fundamental mechanisms that drive LN and identifying new targets for therapeutic intervention are important for treating LN. Emerging evidence indicates that kidney-resident cells in LN are not innocent bystanders but can also play active pathological roles [13, 37]. Podocytes, mesangial cells and RTECs are the most important players. The lack of human LN studies creates a substantial knowledge gap, and our current knowledge of how resident kidney cells contribute to LN is shaped by isolated studies that have not been independently validated. Increasing evidence has shown that RTECs actively participate in the tubulointerstitial pathology of LN through the expression of cytokines, chemokines and fibrogenic molecules. RTEC injury (including tubular atrophy and dilatation) and tubulointerstitial fibrosis have prognostic value for LN and are independent risk factors for LN exacerbation [38]. Renal proximal tubular epithelial cells have high proliferative potential and are necessary for tubular epithelial regeneration and fibrosis progression [39]. RTECs have potential in the diagnosis, prognosis, and treatment of LN, especially when targeting the tubule interstitium in LN patients [40]. The role of RTECs in LN fibrogenesis is an important area of LN-related research [41–43]. In the future, single-cell omics, space transcriptomics and proteolysis targeting chimaeras (PROTAC) could provide systemic tools for advanced LN studies. A single-cell omics study of LN revealed that ubiquitination was widespread during LN disease progression [11]. In addition to the transcriptome, renal spatial proteomics and ubiquitin-modified proteomics could be useful methods for identifying additional changes in the LN microenvironment. There are various cell types in the kidney, which complicates the study of disease mechanisms. In podocytes, inactivation of Hippo maintains podocyte survival and protects renal filtration [44], while inactivation of Hippo in tubules accelerates fibrotic progression in LN. We propose that the Hippo pathway is a double-edged sword in different renal cells associated with autoimmune nephrology. We also examined the expression of SIAH2-LATS2 in the unilateral ureteral obstruction (UUO) fibrosis model, which was associated with much faster fibrosis progression. Firstly, the model was constructed successfully (Fig S6A) and we found that collagen deposits were already produced in large quantities. Proteinuria of UUO mice was also increased rapidly (Figure S6B). Next, we detected the expression of SIAH2 and LATS2 in WT, LN and UUO mice by IHC (Figure S6C). The results showed that SIAH2 was upregulated and LATS2 was downregulated in proximal tubulars in LN and UUO animals. Although LATS2 was not significantly downregulated by UUO compared to LN, which may be related to the activation of apoptosis and structural destruction of renal resident cells in UUO, our finding indicates that the SIAH2-LATS2 was involved in the progression of renal fibrosis caused by different diseases. However, the role of the SIAH2-LATS2 axis in various organ fibrosis needs further verification in specific disease models. Figures and captions were updated in revised manuscript.

Oral administration of vitamin K3 could inactivate YAP and reduce CTGF production, further blocking TGF-β/Hippo crosstalk and thus delaying the exacerbation of fibrosis in mice with LN. We provide new clues for drug treatment of LN fibrosis or ESRD progression other than by targeting the TGF-β pathway. Interestingly, CKD patients are characterized by poor vitamin K status [45]. Reduced recycling of vitamin K was found in rats with CKD, which was likely caused by the reduced activity of g-glutamyl-carboxylase [46]. We found that vitamin K3 exerted its antifibrotic effect by inhibiting the SIAH2-LATS2 axis in LN. Alleviation of RTEC injury protected against the progression of LN fibrosis. Moreover, Garcia et al. reported that nonvalvular atrial fibrillation (NVAF) patients treated with nonvitamin K antagonist oral anticoagulants had a decreased risk and rate of ESRD [47]. This finding indicates that maintaining the level of vitamin K under certain pathological conditions may be beneficial for renal function. This study has several limitations. First, LATS2-KO mice on an MRL/lpr background were not used in this study due to the difficulty of establishing a lupus model. Second, we did not examine the clinical implications of VK3 in trials. Examining the protective effect of VK3 on renal damage would be feasible in an appropriate group of LN patients.

In this study, IHC staining showed that LATS2 was significantly downregulated in LN patient biopsies and mice with LN. Dephosphorylated YAP plays a synergistic transcriptional role in the nucleus, resulting in the upregulation of a series of downstream genes, including CTGF. Restoring LATS2 levels in tubules by expressing exogenous LATS2 or using the SIAH2 inhibitor VK3 could protect renal function and decrease LN fibrosis progression. In summary, our results suggest that SIAH2-LATS2 is a novel target for blocking fibrotic progression in LN.

## Conclusion

We showed the potential of the SIAH2-LATS2 axis as an attractive intervention target in LN and investigated the protective effect of vitamin K3 against LN fibrosis.

### Supplementary Information

**Additional file 1: Supplementary figure 1. **Screenshot of HK-2 cells treated by Anti-dsDNA Ab at different time points.

**Additional file 2: Supplementary figure 2.** A. Body weight change of MRL/lpr mice at different stages. B. Albuminuria and creatinine ratio of MRL/lpr mice at different stages. **C.** Detection of serum anti-dsDNA Ab in MRL/lpr mice at different stages. D. Detection of renal TGF-β in MRL/lpr mice at different stages.

**Additional file 3: Supplementary figure 3.** A. Detection of ANA tiers of MRL/lpr mice at different stages (scale bar, 100μm). B. Detection of renal IgG immune deposition in MRL/lpr mice at different stages (scale bar, 5μm). C, Co-localization analysis of LATS2 and CTGF in LN (scale bar,5μm).

**Additional file 4: Supplementary figure 4.** A. HK-2 cells expressed exogenous LATS2 after infected with Ad-LATS2 in concentration dependent. B. Illustration of renal in situ injection. C-D. Western blot and RT-qPCR analysis of MRL/lpr mice infected by Ad-LATS2 after 10 days. E. 24 h proteinuria detection of MRL/lpr mice infected by Ad-LATS2. F. qPCR analysis of renal proximal IL-6, IFN-γ and α-SMA expression.

**Additional file 5: Supplementary figure 5.** A, The LATS2 mRNA was not changed in LN mice. B, The mRNA expression of LATS2 in LN patients’ tubulars. Data source GSE127797, scripts is available upon request. C, Overexpression of LATS2 reduced CTGF production induced by TGF-β in HK-2 cells. D, Upregulated SIAH2 and E, Zyxin mRNA after TGF-β treatment. F, Western blot analysis of mutant SIAH2-LATS2 interaction and (G) H150A-SIAH2, K672R LATS2. H, Expression of LATS2 analyzed by IHC staining, vitamin K3 restored the LATS2 levels (scale bar, 40μm).

**Additional file 6: Supplementary figure 6.** A, HE, Sirus red and Masson staining analysis of UUO, semi-quantitative analysis of tubular injury scores and collagen area were performed (*n*=6). B, Proteinuria analysis of UUO mice in one week (*n*=6). C, Analysis of SIAH2 and LATS2 expression in WT, LN and UUO group (scale bar, 20μm).

**Additional file 7: Supplementary Table 1. **qPCR primers used in this study.** Supplementary Table 2. **Predicted sequences of SIAH2 promoter bind to RUNX1 (JASPAR, https://www.jaspar.genereg.net/). **Supplementary Table 3. **Clinical information of lupus nephritis patients (renal biopsies).

**Additional file 8.**

## Data Availability

No datasets were generated or analysed during the current study.

## Abbreviations

LN: Lupus nephritis
SLE: Systemic lupus erythematosus
CKD: Chronic kidney disease
ESRD: End stage of renal disease
CTGF: Connective tissue growth factor
IHC: Immunohistochemistry
LATS2: Large tumor suppressor kinase 2
SIAH2: Seven in absentia homologs 2
RTECs: Renal tubular epithelial cells
UUO: Unilateral ureteral obstruction
