# Supplementary figures and images for: LATS2 degradation promoted fibrosis damage and rescued by vitamin K3 in lupus nephritis
Source: Arthritis Res Ther. 2024 Mar 9;26:64. doi: 10.1186/s13075-024-03292-y (PMC10924340; doi:10.1186/s13075-024-03292-y)

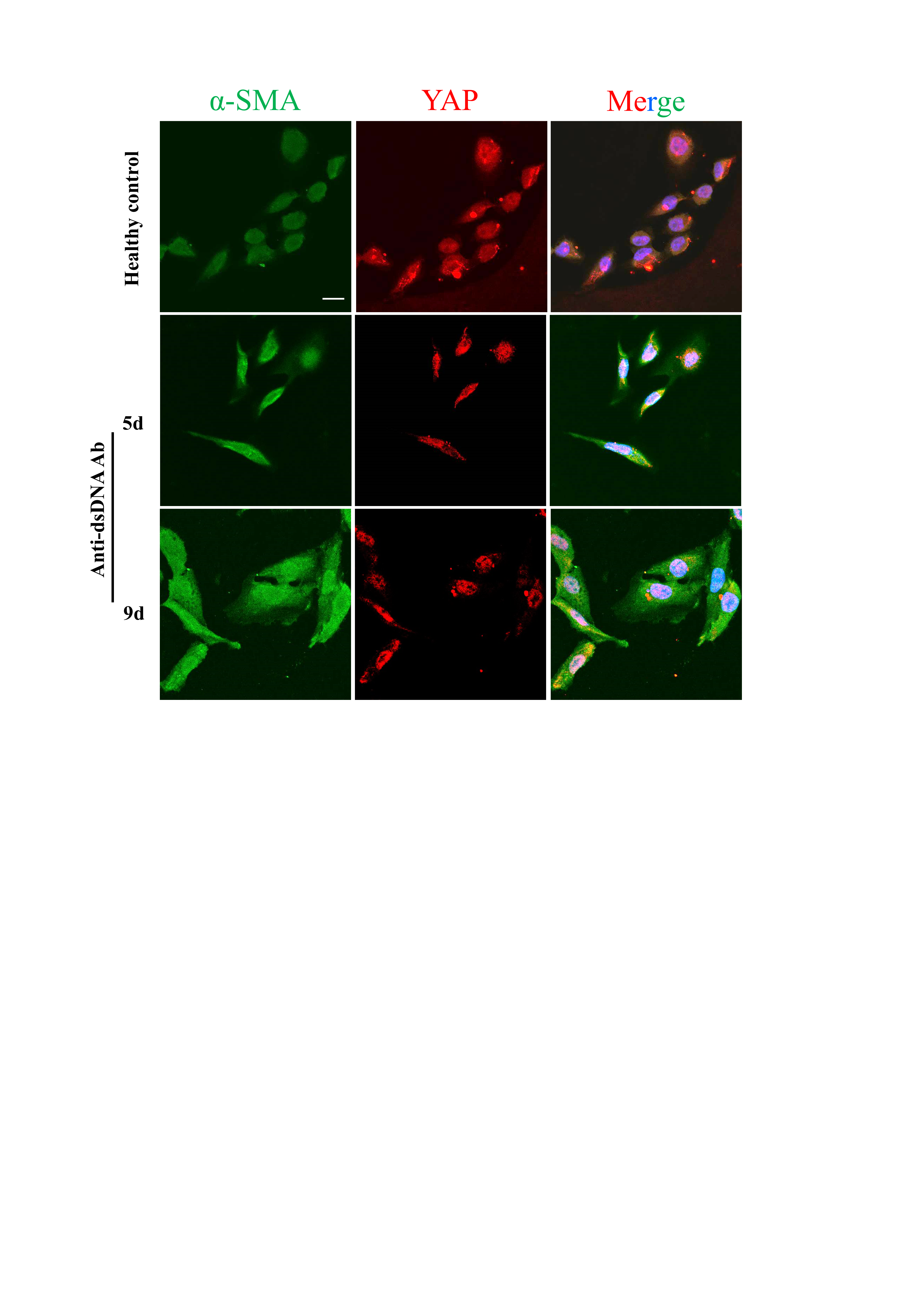

Supplement: Supplementary file 1 — Additional file 1: Supplementary figure 1. Screenshot of HK-2 cells treated by Anti-dsDNA Ab at different time points. [file 13075_2024_3292_MOESM1_ESM.tif]

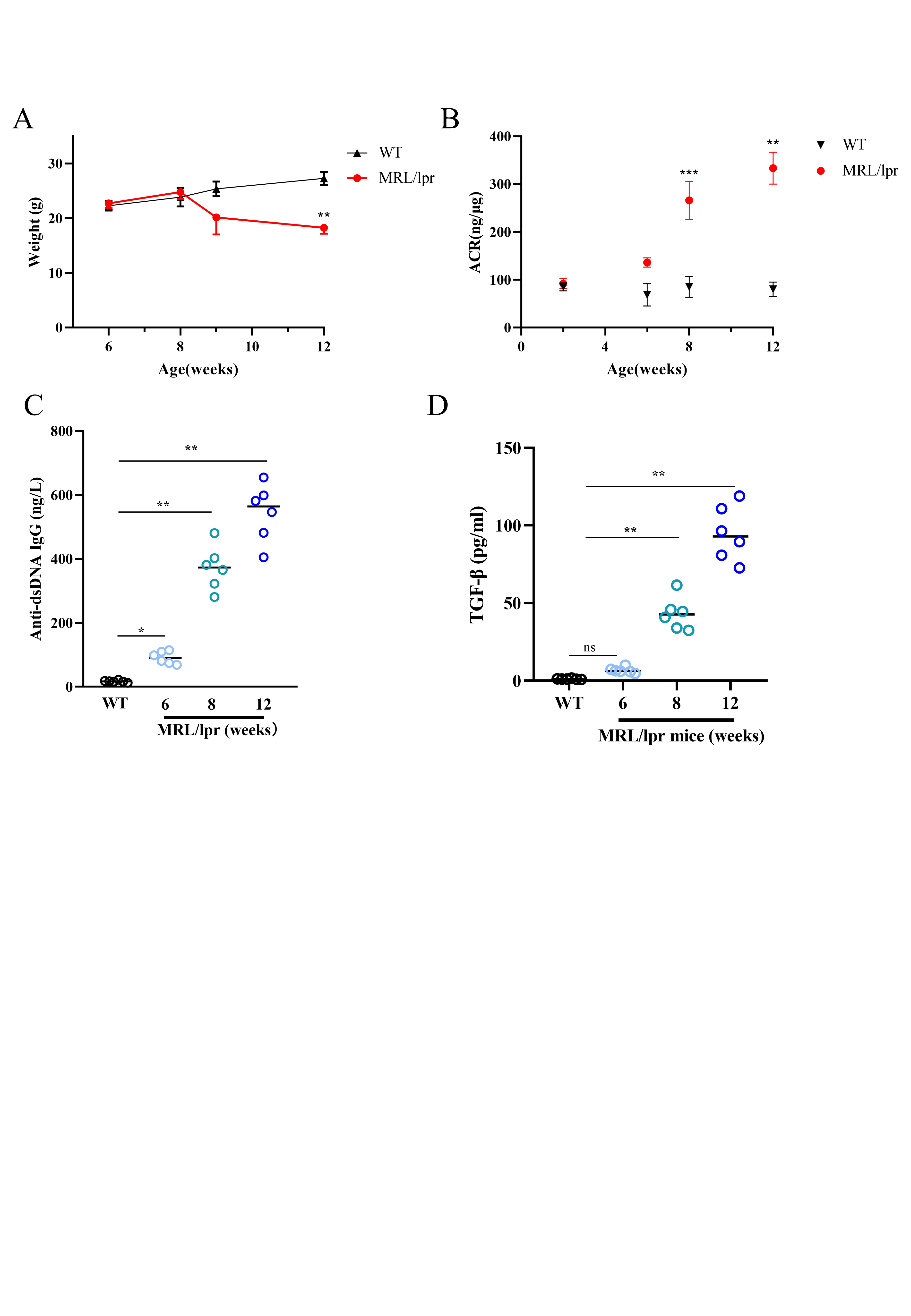

Supplement: Supplementary file 2 — Additional file 2: Supplementary figure 2. A. Body weight change of MRL/lpr mice at different stages. B. Albuminuria and creatinine ratio of MRL/lpr mice at different stages. C. Detection of serum anti-dsDNA Ab in MRL/lpr mice at different stages. D. Detection of renal TGF-β in MRL/lpr mice at different stages. [file 13075_2024_3292_MOESM2_ESM.tif]

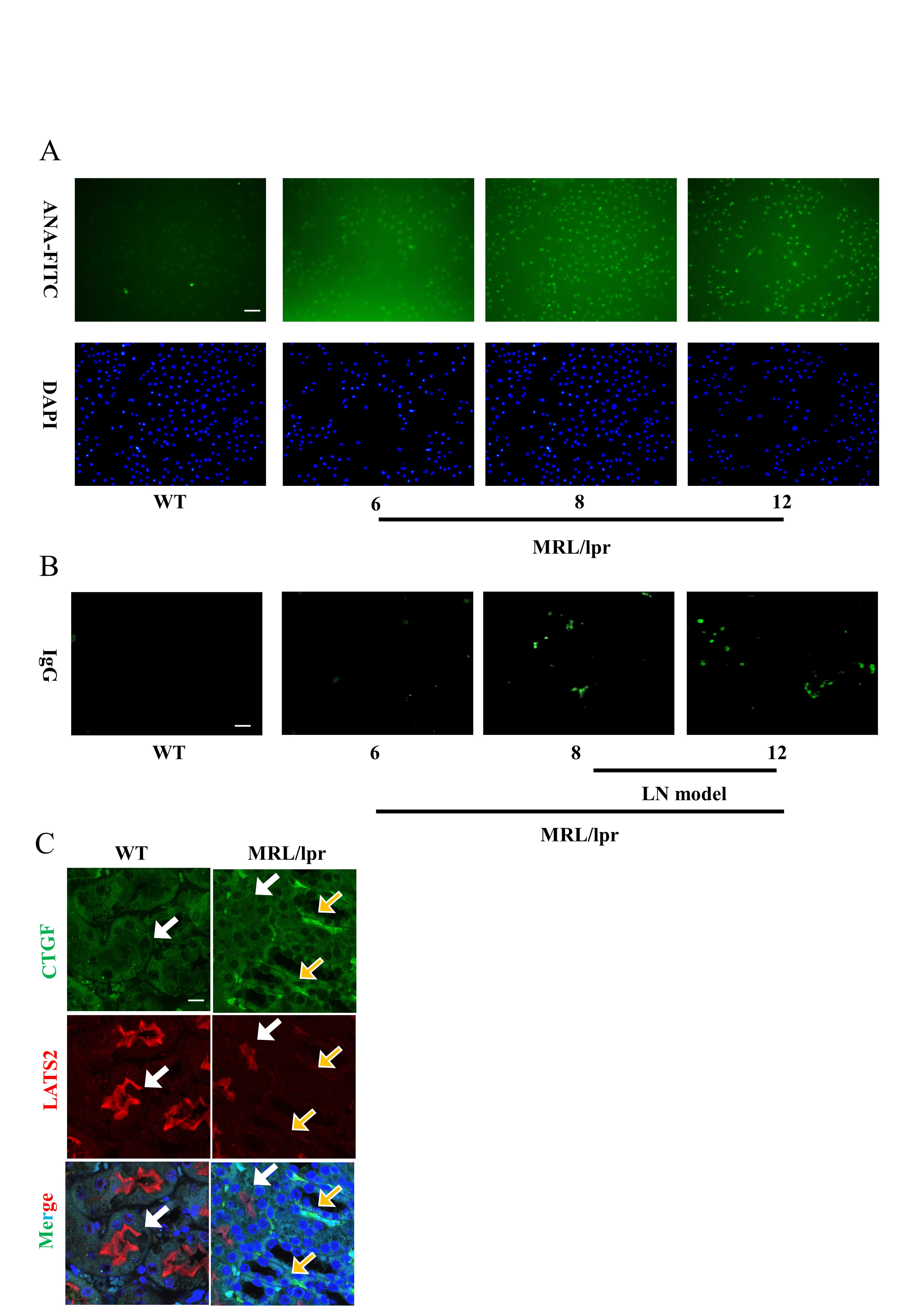

Supplement: Supplementary file 3 — Additional file 3: Supplementary figure 3. A. Detection of ANA tiers of MRL/lpr mice at different stages (scale bar, 100μm). B. Detection of renal IgG immune deposition in MRL/lpr mice at different stages (scale bar, 5μm). C, Co-localization analysis of LATS2 and CTGF in LN (scale bar,5μm). [file 13075_2024_3292_MOESM3_ESM.tif]

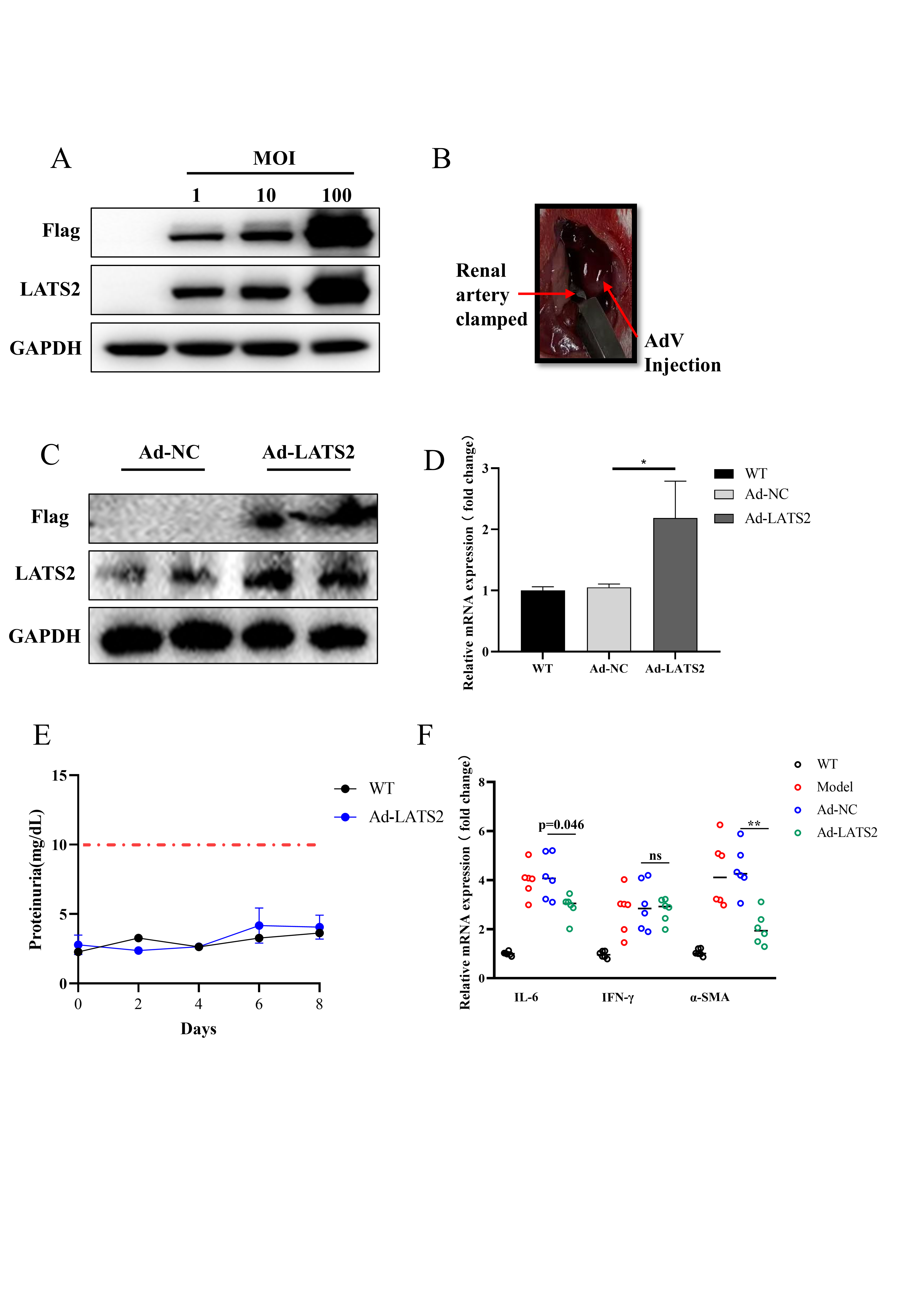

Supplement: Supplementary file 4 — Additional file 4: Supplementary figure 4. A. HK-2 cells expressed exogenous LATS2 after infected with Ad-LATS2 in concentration dependent. B. Illustration of renal in situ injection. C-D. Western blot and RT-qPCR analysis of MRL/lpr mice infected by Ad-LATS2 after 10 days. E. 24 h proteinuria detection of MRL/lpr mice infected by Ad-LATS2. F. qPCR analysis of renal proximal IL-6, IFN-γ and α-SMA expression. [file 13075_2024_3292_MOESM4_ESM.tif]

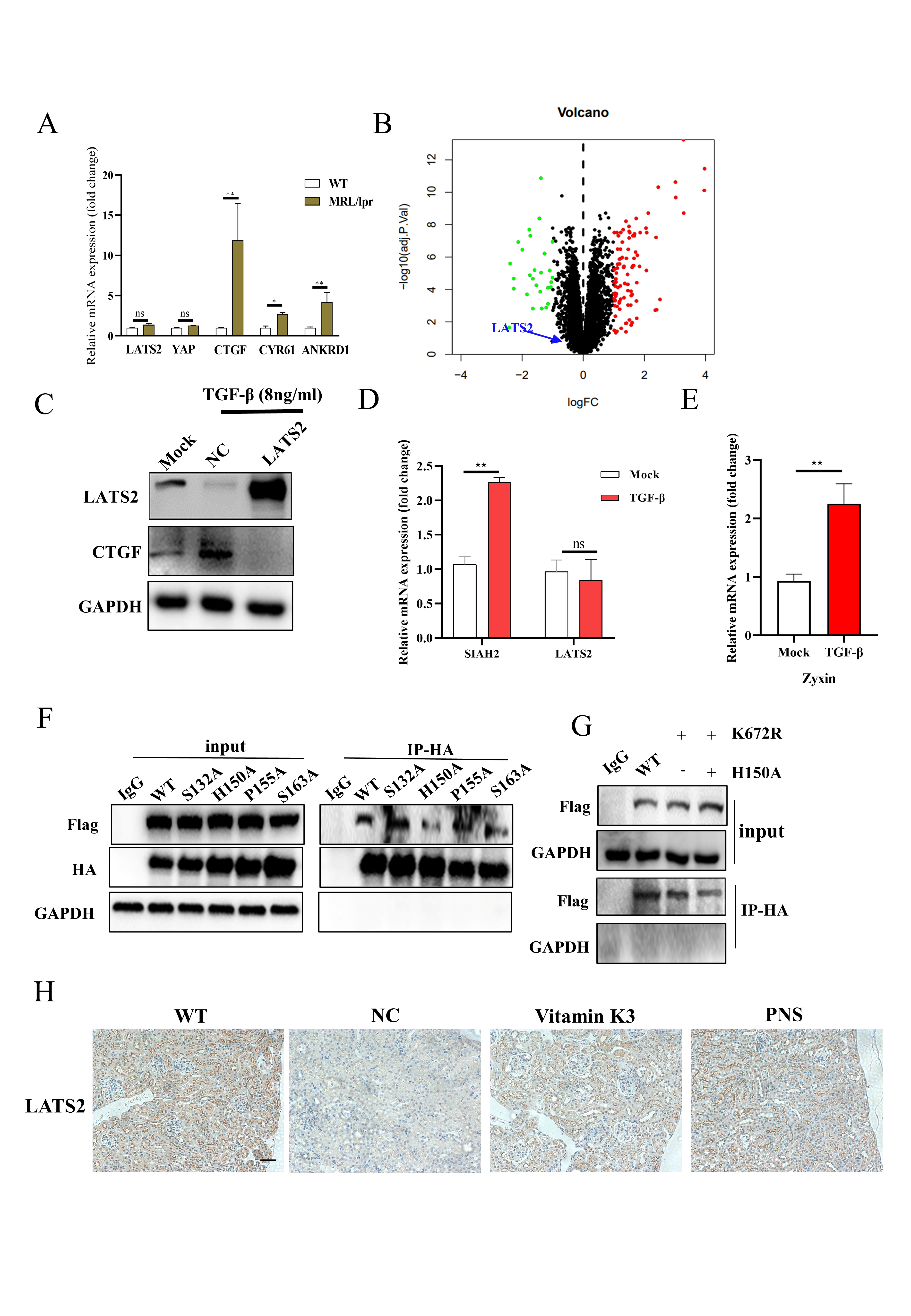

Supplement: Supplementary file 5 — Additional file 5: Supplementary figure 5. A, The LATS2 mRNA was not changed in LN mice. B, The mRNA expression of LATS2 in LN patients’ tubulars. Data source GSE127797, scripts is available upon request. C, Overexpression of LATS2 reduced CTGF production induced by TGF-β in HK-2 cells. D, Upregulated SIAH2 and E, Zyxin mRNA after TGF-β treatment. F, Western blot analysis of mutant SIAH2-LATS2 interaction and (G) H150A-SIAH2, K672R LATS2. H, Expression of LATS2 analyzed by IHC staining, vitamin K3 restored the LATS2 levels (scale bar, 40μm). [file 13075_2024_3292_MOESM5_ESM.tif]

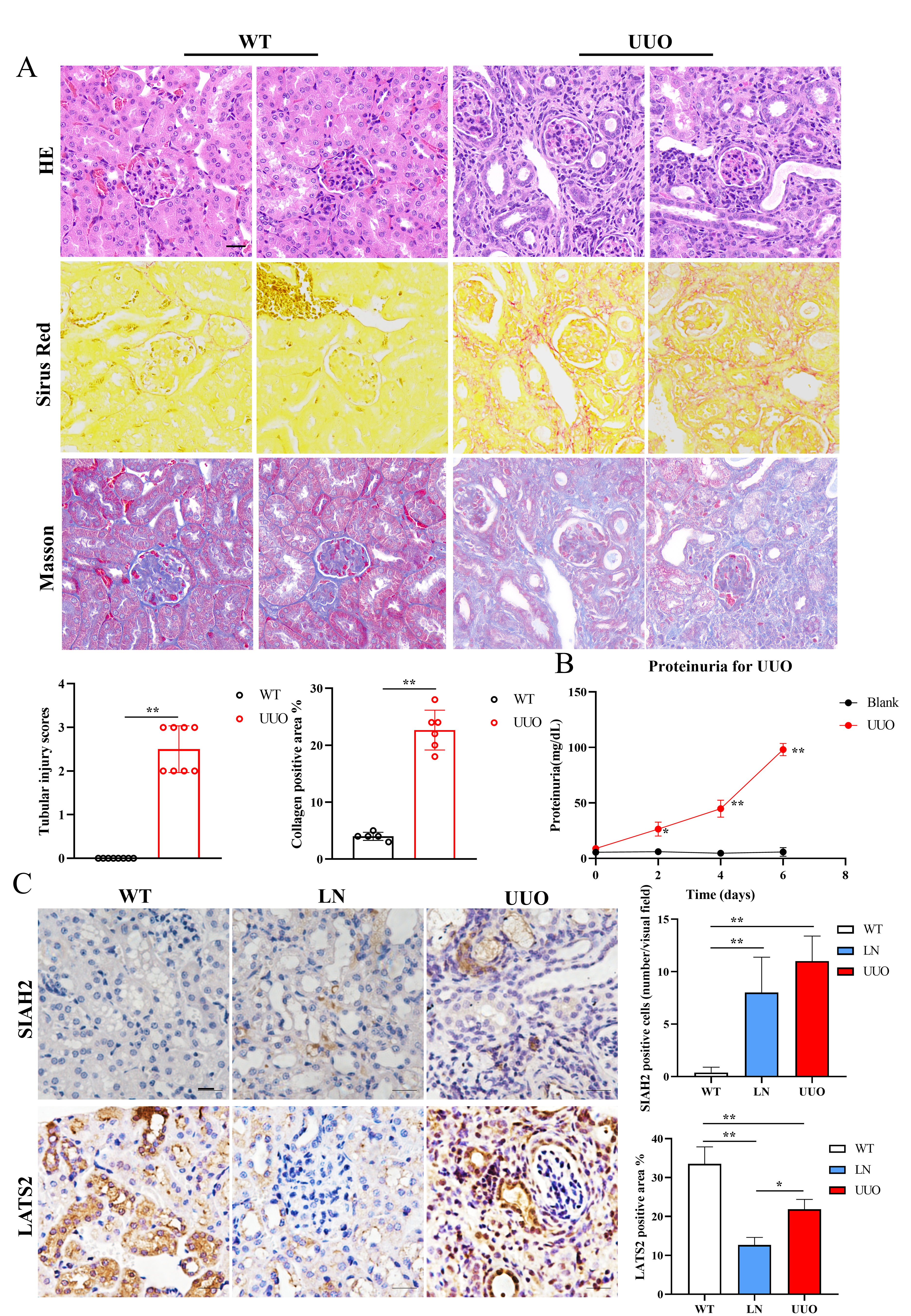

Supplement: Supplementary file 6 — Additional file 6: Supplementary figure 6. A, HE, Sirus red and Masson staining analysis of UUO, semi-quantitative analysis of tubular injury scores and collagen area were performed (n=6). B, Proteinuria analysis of UUO mice in one week (n=6). C, Analysis of SIAH2 and LATS2 expression in WT, LN and UUO group (scale bar, 20μm). [file 13075_2024_3292_MOESM6_ESM.tif]
