# Supplementary material for: LATS2 degradation promoted fibrosis damage and rescued by vitamin K3 in lupus nephritis
Source: Arthritis Res Ther. 2024 Mar 9;26:64. doi: 10.1186/s13075-024-03292-y (PMC10924340; doi:10.1186/s13075-024-03292-y)
Supplement: Supplementary file 7 — Additional file 7: Supplementary Table 1. qPCR primers used in this study. Supplementary Table 2. Predicted sequences of SIAH2 promoter bind to RUNX1 (JASPAR, https://www.jaspar.genereg.net/). Supplementary Table 3. Clinical information of lupus nephritis patients (renal biopsies). [file 13075_2024_3292_MOESM7_ESM.docx]

**Supplementary Table 1 Primers used for RT-qPCR in this study.**

| **Human** | **Forward** | **Reverse** |
| --- | --- | --- |
| GAPDH | TGTTCGTCATGGGTGTGAAC | GTTGTCATGGATGACCTTGG |
| LATS2 | CAGGATGCGACCAGGAGATG | CCGCACAATCTGCTCATTC |
| αSMA | GGGACGACATGGAAAAGATCTG | CAGGGTGGGATGCTCTTCA |
| Collagen I | CAGGGTGGGATGCTCTTCA | CCGCCATACTCGAACTGGAA |
| CTGF | ACCGACTGGAAGACACGTTTG | CCAGGTCAGCTTCGCAAGG |
| CYR61 | GGTCAAAGTTACCGGGCAGT | GGAGGCATCGAATCCCAGC |
| ANKRD1 | AGACCTCAACGCCAAAGACA | CTTGATGTTGAGATCCGCGC |
| YAP | CCGCACAATCTGCTCATTC | GATAGCAGGGCGTGAGGAAC |
| SIAH2 | CGCCAGAAGTTGAGCTGCT | TGGTGGCATACTTACAGGGAA |
| **Mice** | **Forward** | **Reverse** |
| GAPDH | TATGACTCTACCCACGGCAAG | TACTCAGCACCAGCATCACC |
| LATS2 | ATCCTCCCAAAGGGTACAGCACAG | TGGTGGCGTCTTGTTCTGGAAG |
| αSMA | CAGGGAGTAATGGTTGGAAT | TCTCAAACATAATCTGGGTCA |
| Collagen I | AAGGGGTCTTCCTGGTGAAT | GGGGTACCACGTTCTCCTC |
| CTGF | TGTGCACTGCCAAAGATGGTGCAC | TGGGCAGGCGCACGTCCATG |
| CYR61 | GAGGCTTCCTGTCTTTGGCAC | ACTCTGGGTTGTCATTGGTAAC |
| TGFβ1 | CACGTGGAGCTGTACCAGAA | GAACCCGTTGATGTCCACTT |
| YAP | AAATGCTCCAAAATGTCAGGA | CATTCGGAGTCCCTCCATC |
| IL-6 | TCTTGGGACTGATGCTGGTGA | GCAAGTGCATCATCGTTGTTCA |
| IFN-γ | TACACACTGCATCTTGGCTTTG | CTTCCACATCTATGCACTTGAG |

**Supplementary Table 2 Potential binding sites of SIAH2 promoter with RUNX1.**

| Name | Score | Relative score | Start | End | Strand | Sequence |
| --- | --- | --- | --- | --- | --- | --- |
| MA0002.2.Runx1 | 9.877388 | 0.890679749 | 403 | 413 | + | CTCTGTGCTTT*^1^ |
| MA0002.2.Runx1 | 9.161777 | 0.866737885 | 267 | 277 | + | TACAGTGGTTT*^2^ |
| MA0002.2.Runx1 | 9.104003 | 0.865241461 | 286 | 296 | + | CATTGAGGTAA |
| MA0002.2.Runx1 | 7.672184 | 0.847686268 | 1724 | 1734 | + | GGGTGTGGCCA |
| MA0002.2.Runx1 | 7.596475 | 0.84621021 | 1506 | 1516 | - | TTCTGTGTTCG |
| MA0002.2.Runx1 | 7.561121 | 0.845520933 | 1777 | 1787 | - | TTTTCTGGGTT |
| MA0002.2.Runx1 | 7.066123 | 0.835870267 | 234 | 244 | + | TGTTGTGTGTT |
| MA0002.2.Runx1 | 6.664811 | 0.82804615 | 236 | 246 | + | TTGTGTGTTCT |
| MA0002.2.Runx1 | 6.479089 | 0.824425238 | 940 | 950 | + | CTCTGTGTCTC |
| MA0002.2.Runx1 | 6.367117 | 0.822242202 | 334 | 344 | - | GGGTGTGGCGT |

* Site identified in this study.

**Supplementary Table 3 Information of lupus nephritis patients.**

| Patients | Age | Gender | Anti-dsDNA-Ab (IU/ml) | Anti-nuclear-Ab (titer) | Anti-Sm-Ab | Urinary  microalbumin  (mg/L) | LN (Stage/Class) |
| --- | --- | --- | --- | --- | --- | --- | --- |
| 1 | 34 | Female | 178 | 160 | (-) | 235 | III |
| 2 | 32 | Female | ＞300 | 160 | (-) | 140 | IV |
| 3 | 30 | Female | ＞300 | ＞500 | (-) | 258 | IV plus V |
| 4 | 36 | Female | 155 | ＞500 | (+) | 1208 | III |
| 5 | 27 | Female | 41 | 316 | (-) | 910 | IV |
| 6 | 21 | Female | 118 | 227 | (-) | 1198 | IV plus V |
| 7 | 44 | Female | ＞300 | 422 | (+) | 2506 | IV |
| 8 | 20 | Female | 261 | 316 | (-) | 1976 | IV |
