# Supplementary material for: LATS2 degradation promoted fibrosis damage and rescued by vitamin K3 in lupus nephritis
Source: Arthritis Res Ther. 2024 Mar 9;26:64. doi: 10.1186/s13075-024-03292-y (PMC10924340; doi:10.1186/s13075-024-03292-y)

Figure 1B

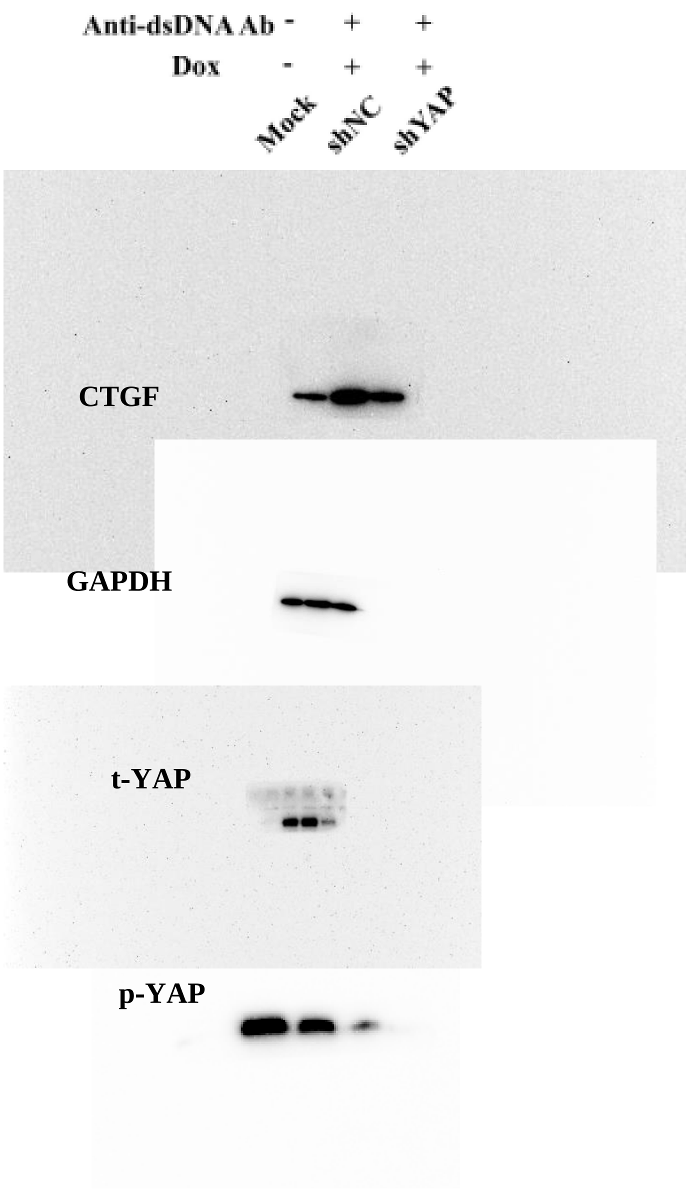

Figure 3A

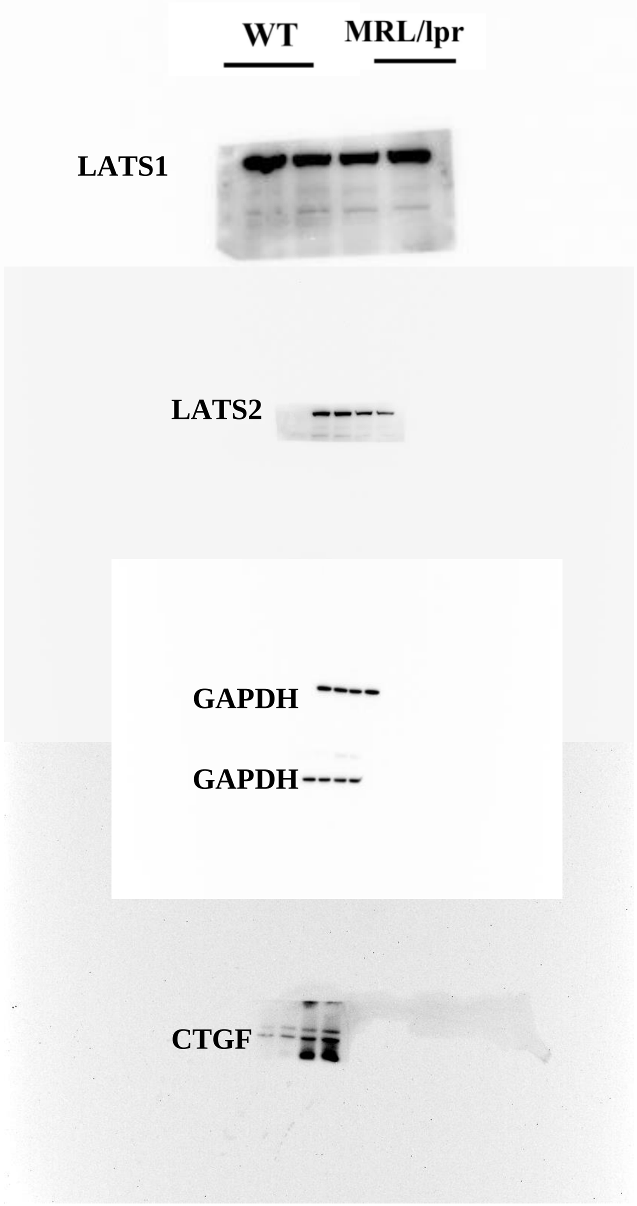

Figure 3B

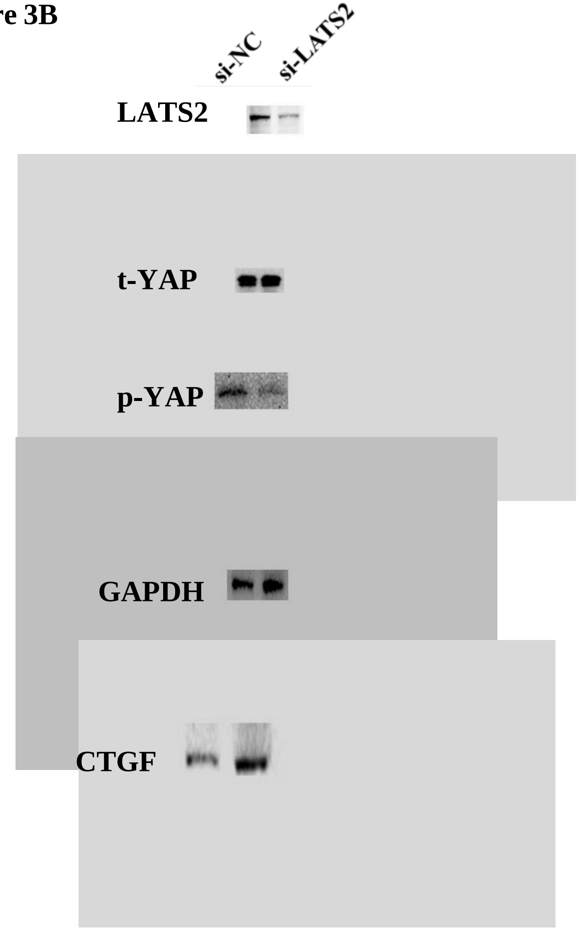

Figure 3D

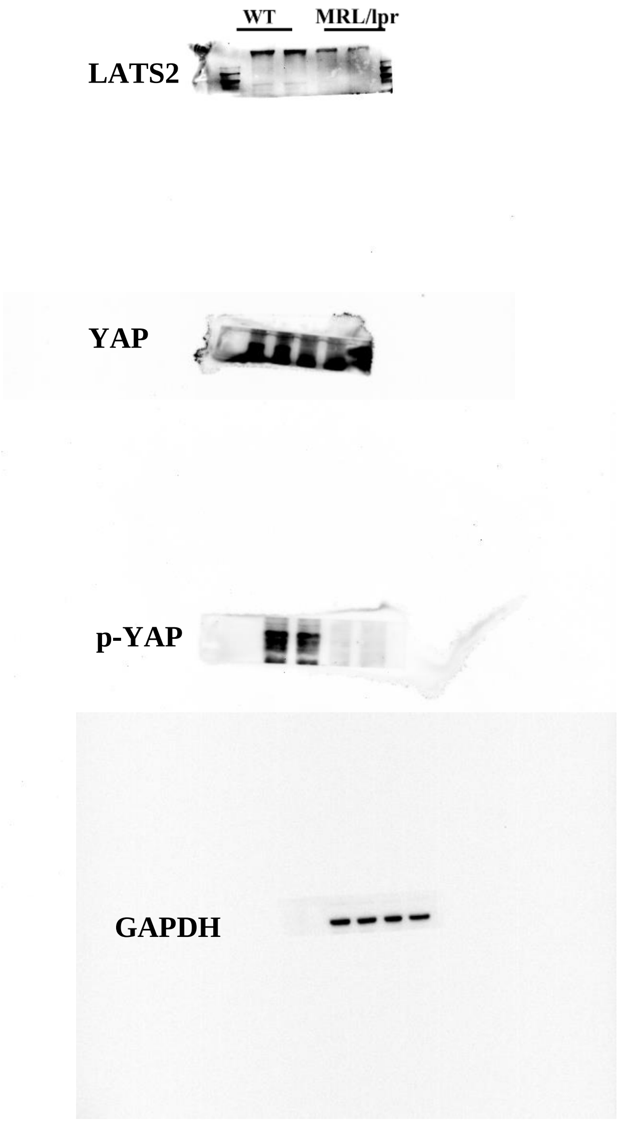

Figure 4C

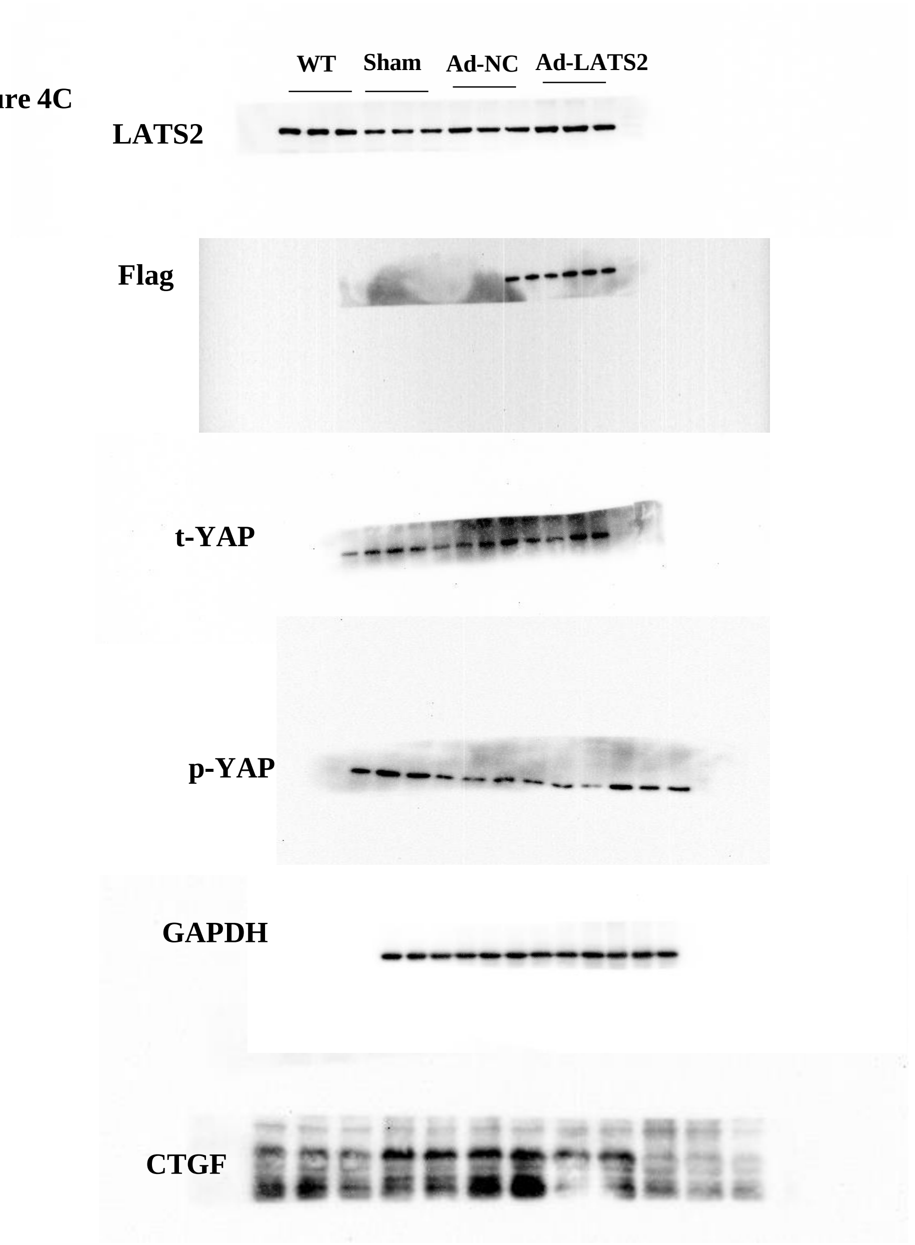

Figure 5B

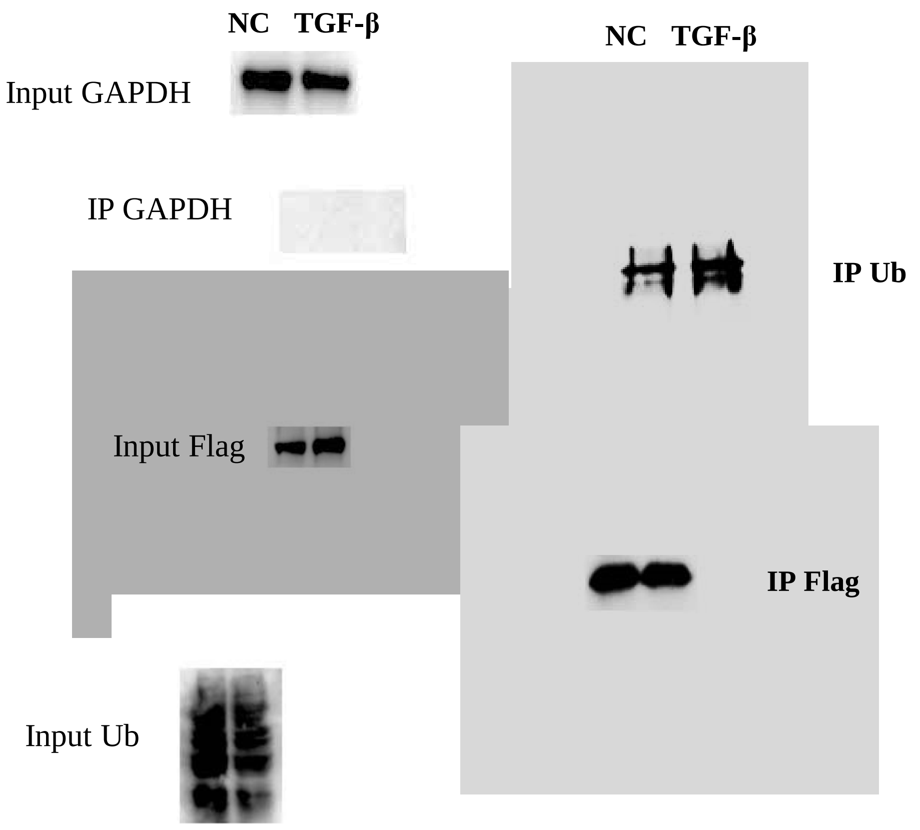

Figure 5C

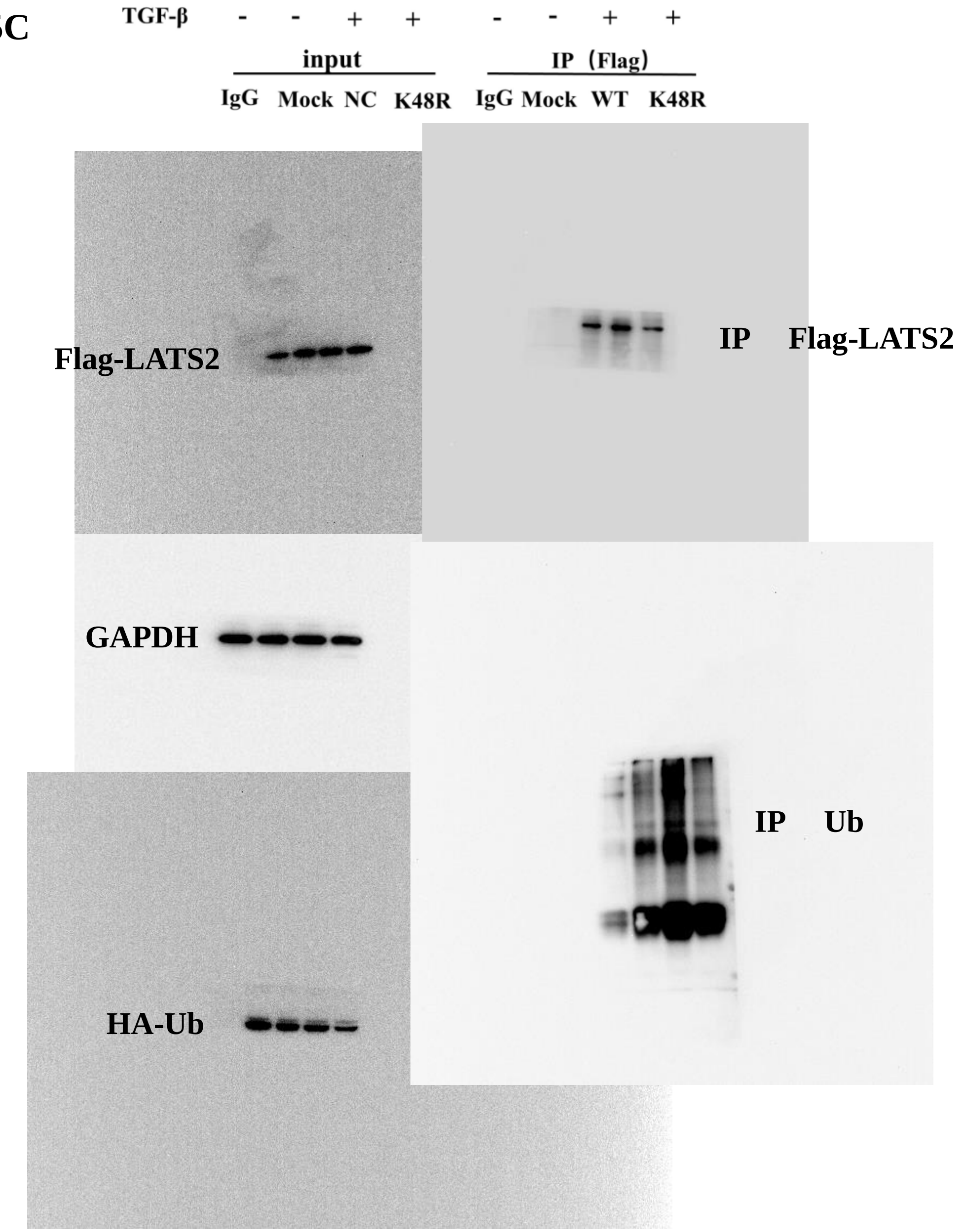

Figure 5F

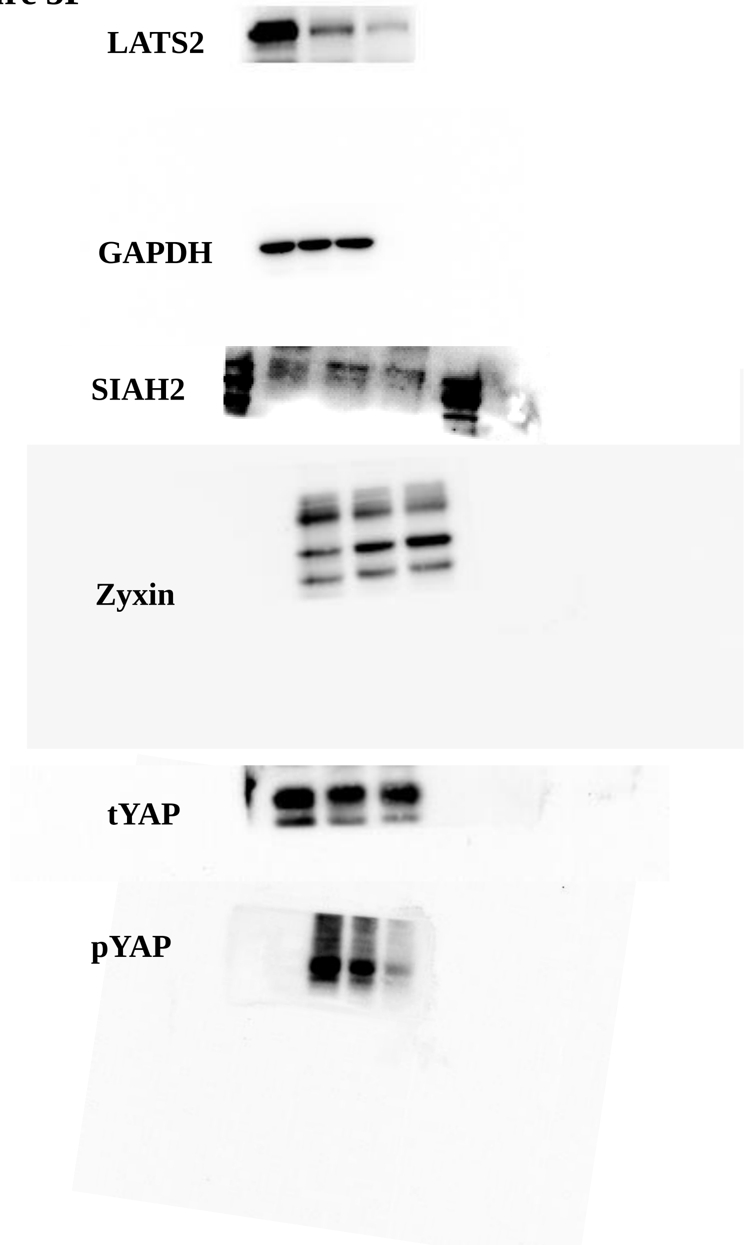

Figure 5H

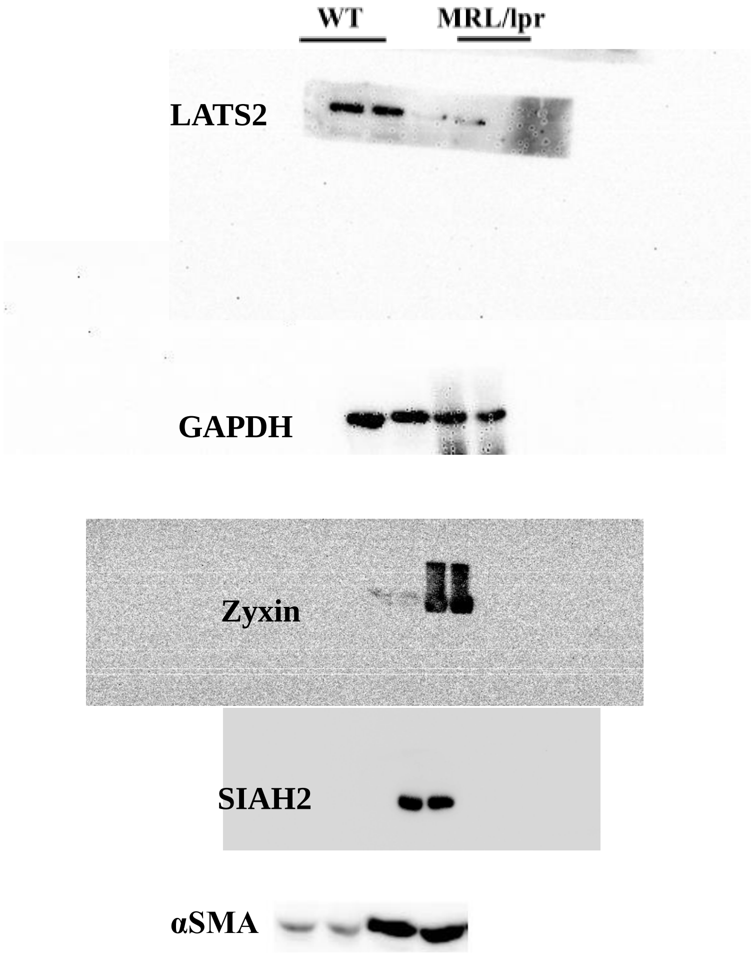

Figure 5I

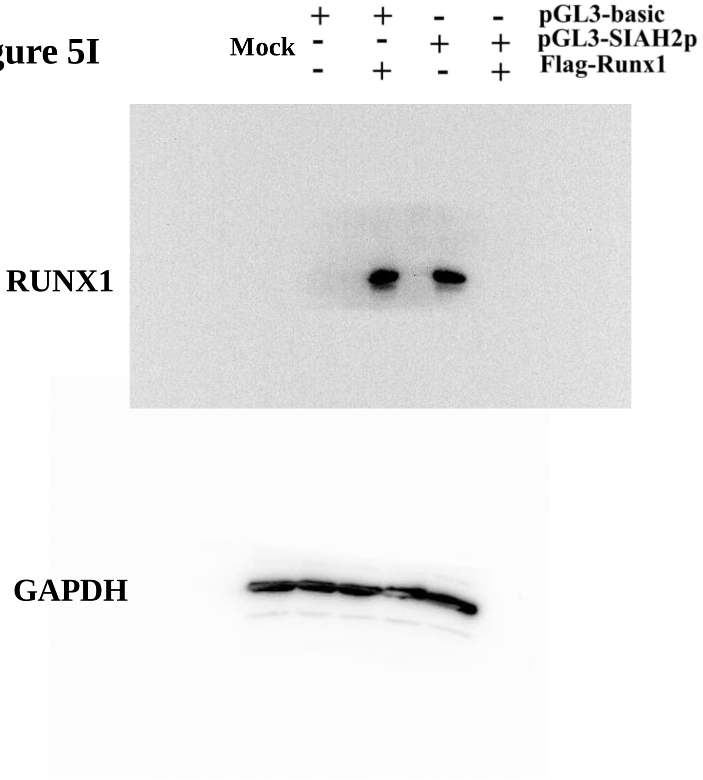

Figure 6A

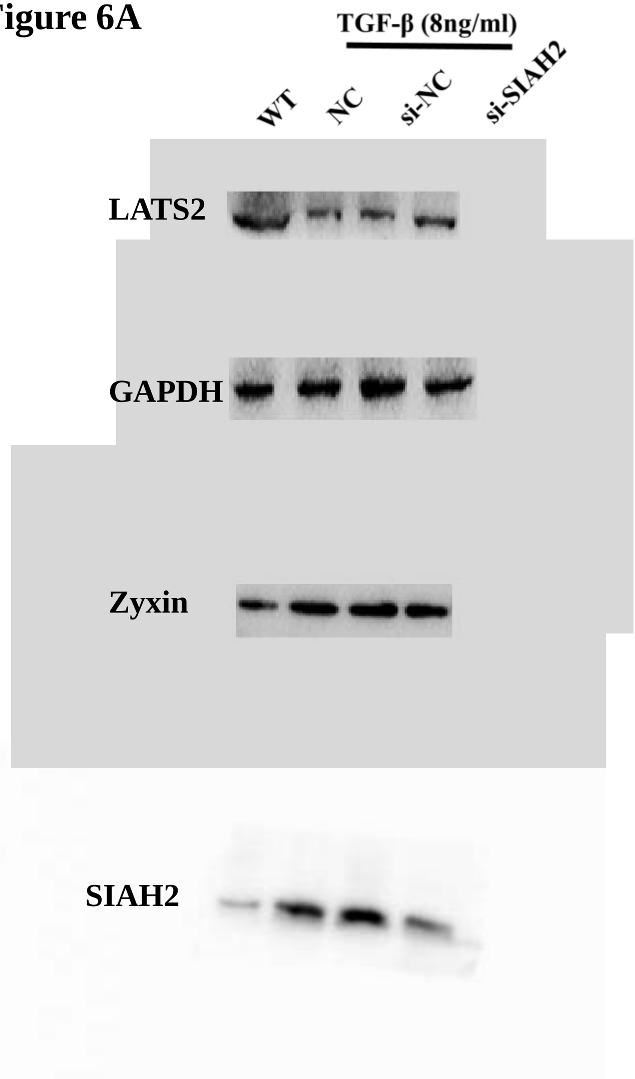

Figure 6C

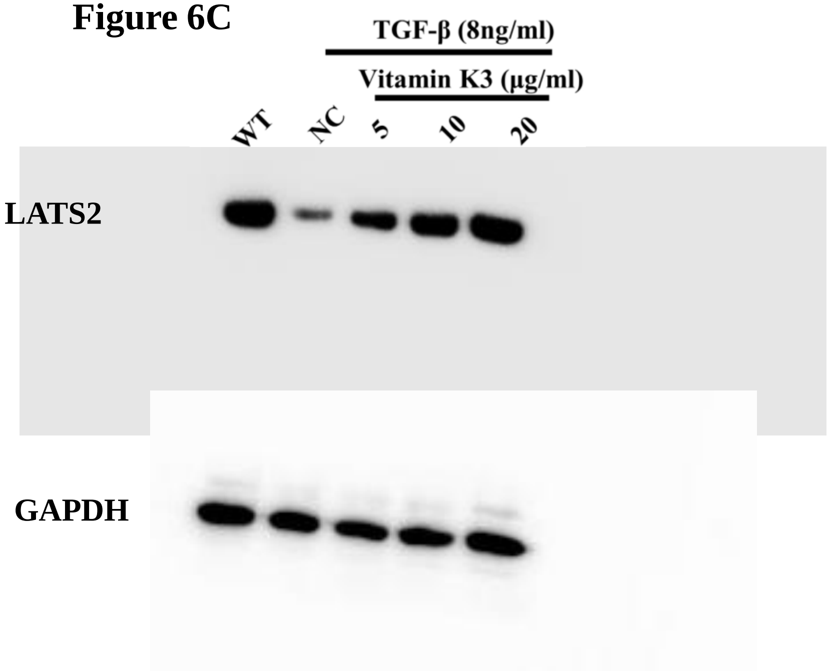

Figure 7A

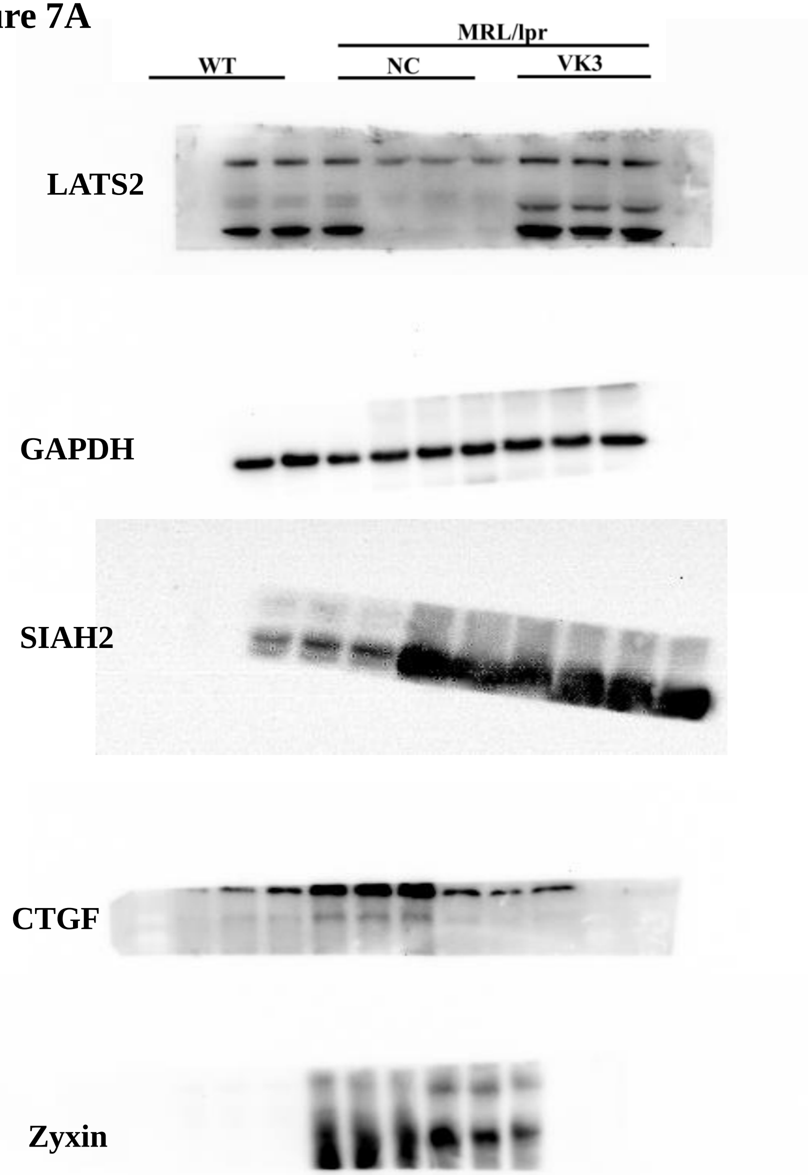

Supplement: Supplementary file 8 — Additional file 8. [file 13075_2024_3292_MOESM8_ESM.pdf]
